# Chirality-assisted lateral momentum transfer for bidirectional enantioselective separation

**DOI:** 10.1038/s41377-020-0293-0

**Published:** 2020-04-16

**Authors:** Yuzhi Shi, Tongtong Zhu, Tianhang Zhang, Alfredo Mazzulla, Din Ping Tsai, Weiqiang Ding, Ai Qun Liu, Gabriella Cipparrone, Juan José Sáenz, Cheng-Wei Qiu

**Affiliations:** 1 School of Mechanical Engineering, Xi’an Jiaotong University, Xi’an, 710049 China; 2 School of Electrical and Electronic Engineering, Nanyang Technological University, Singapore, 639798 Singapore; 3 Department of Electrical and Computer Engineering, National University of Singapore, Singapore, 117583 Singapore; 4 School of Optoelectronic Engineering and Instrumentation Science, Dalian University of Technology, Dalian, 116024 China; 5 School of Physics, Harbin Institute of Technology, Harbin, 150001 China; 6 CNR-NANOTEC, LiCryL and Centre of Excellence CEMIF. CAL, Ponte P. Bucci, Cubo 33B, 87036 Rende (CS), Italy; 7 Department of Electronic and Information Engineering, The Hong Kong Polytechnic University, Hung Hom, Kowloon, Hong Kong China; 8 Department of Physics, University of Calabria, Ponte P. Bucci, Cubo 33B, 87036 Rende (CS), Italy; 9 Donostia International Physics Center, 20018 Donostia-San Sebastián, Spain

**Keywords:** Applied optics, Optical manipulation and tweezers

## Abstract

Lateral optical forces induced by linearly polarized laser beams have been predicted to deflect dipolar particles with opposite chiralities toward opposite transversal directions. These “chirality-dependent” forces can offer new possibilities for passive all-optical enantioselective sorting of chiral particles, which is essential to the nanoscience and drug industries. However, previous chiral sorting experiments focused on large particles with diameters in the geometrical-optics regime. Here, we demonstrate, for the first time, the robust sorting of Mie (size ~ wavelength) chiral particles with different handedness at an air–water interface using optical lateral forces induced by a single linearly polarized laser beam. The nontrivial physical interactions underlying these chirality-dependent forces distinctly differ from those predicted for dipolar or geometrical-optics particles. The lateral forces emerge from a complex interplay between the light polarization, lateral momentum enhancement, and out-of-plane light refraction at the particle-water interface. The sign of the lateral force could be reversed by changing the particle size, incident angle, and polarization of the obliquely incident light.

## Optical sorting: Forcing mirror-image microparticles to go their own way

A setup that uses lasers to automatically sort compounds with nearly imperceptible chemical differences could improve the efficiency of drug manufacturing. Many pharmaceuticals are difficult to purify because they are chiral, meaning they exist as one of two mirror-image structures that rotate in opposite directions under linearly polarized light. Cheng-Wei Qiu from the National University of Singapore and colleagues now report that lateral forces generated by a mix of reflected and refracted light rays can separate chiral microspheres floating on the surface of water. The team developed a line-shaped optical trap to hold a mix of chiral particles in place, and then directed a polarized laser beam into the trap. Microscopic imaging revealed that particles with identical chirality moved laterally when exposed to the beam, with velocities dependent on incident angle and microparticle size.

## Introduction

Enantiomer sorting has attracted tremendous attention owing to its significant applications in both material science and the drug industry^1–5^. In 2006, 80% of drugs approved by the FDA (U.S. Food and Drug Administration) were chiral^6,7^. Among them, 75% were single enantiomers. Recently, optical enantioseparation has attracted much attention owing to the emergence of optical phenomena^8–13^. Unstructured, plane-wave-like light fields can induce optical lateral forces on appropriately shaped objects as an optical analogue to aerodynamic lift^14^. Circularly polarized (CP) beams can induce spin-dependent lateral forces on achiral spherical particles when they are placed near an interface^15,16^. The displacements of particles controlled by the spin of the light can be perpendicular to the direction of the light beam^17–19^. Only a few experimental observations of spin-dependent lateral forces have hitherto been reported. These lateral forces, associated with optical spin–orbit interactions, differ from the “chirality-dependent” lateral forces induced by linearly polarized beams, which deflect dipolar chiral particles with opposite handedness towards opposite lateral directions^20–24^. Most examples of optical lateral forces induced by chirality are only theoretical predictions based on dipole (radius ≤ 50 nm) or geometrical-optics (e.g., radius > 10 µm) particles under the illumination of beams with intensity gradients^25,26^. Meanwhile, the chiral particles used in reported experiments are tens of micrometers in size, in the geometrical-optics regime, where the mechanism and methodology are quite different from the dipole approximation and Mie theories.

Chirality-dependent lateral forces have been theoretically proposed to be powerful tools for all-optical enantiomer sorting. Most reported methods are only theoretical models based on the analogous photogalvanic effect^27^, Stern–Gerlach-type deflectors^26,28^, standing waves^23^, and plasmonic nanoapertures^29–31^. Experiments on enantioselective optical forces include the use of atomic force microscopy (AFM)^30^ and helicity-dependent optical forces^25,26,32–34^. The helicity-dependent optical forces require two counterpropagating beams with opposite helicities. These experiments exploring the interactions of light helicity and particle chirality do not belong to the field of optical lateral forces because they are not applicable to linearly polarized beams (see Supplementary Fig. S1). The system with two counterpropagating helical beams also has difficulties in the manipulation of particles smaller than 2 µm. Despite potential applications, there has been no experimental evidence of chirality-dependent lateral forces induced by a single, non-gradient plane wave on a Mie (radius ~ wavelength) chiral particle.

## Results

### Principle of the optical lateral force on Mie chiral particles

**Fig. 1:**
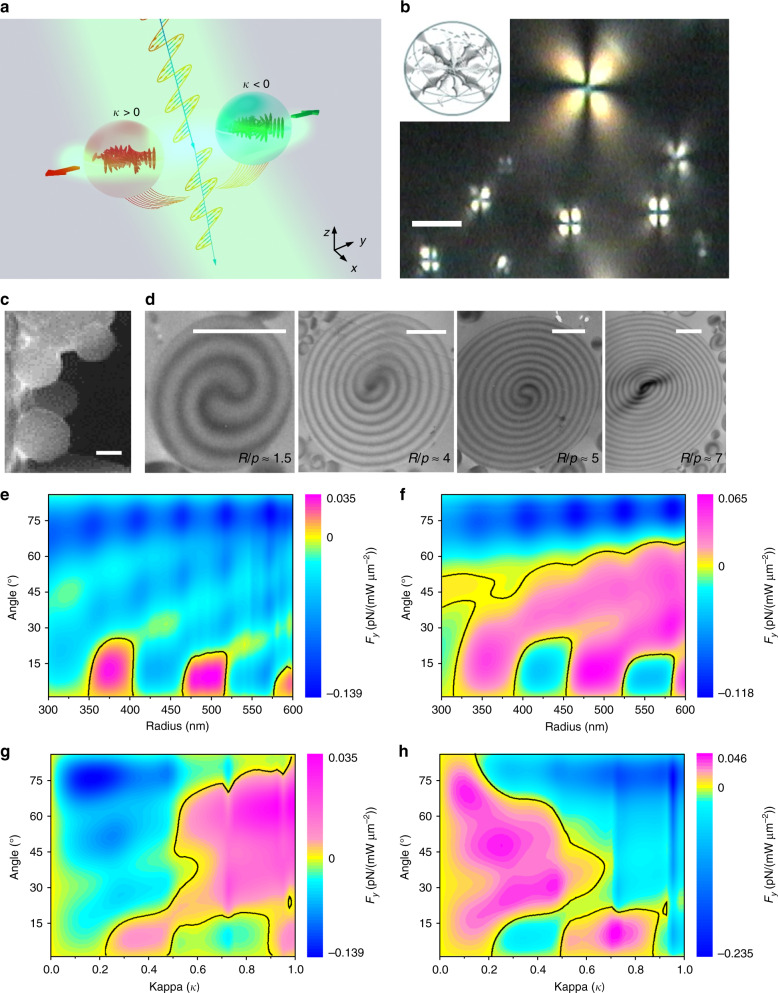
Reversible optical lateral forces for bidirectional sorting of chiral particles. **a** Illustration of the sorting of chiral particles with different handedness (*κ*) by the optical lateral force at an air–water interface. **b** Microscopic image of the cholesteric polymerized microparticles between crossed polarizers. The scale bar equals 5 µm. **c** SEM image of dry polymeric microparticles. The scale bar equals 1 µm. **d** TEM images of thin slices of microparticles with increasing *R*/*p*, in the range of 1 < *R*/*p* < 8. The scale bars equal 1 µm. **e**, **f** Variation in the optical lateral force with particle size and incident angle under the illumination of s- (**e**) and p- (**f**) polarized beams. **g**, **h** Reversible optical lateral force arising from the effect of kappa under the illumination of s- (**g**) and p- (**h**) polarized beams. The radii used in **g**, **h** are 500 nm

Cholesteric polymerized microparticles^35^ floating at an air–water interface provide a suitable model system to experimentally investigate chirality-dependent optical lateral forces (see Fig. 1a). Chiral particles with different handedness *κ* > 0 and *κ* < 0, under the illumination of an s-polarized beam with incident angle *θ*, experience optical lateral forces to the left (*F*_*y*_ < 0) or right (*F*_*y*_ > 0), respectively. The chirality parameter *κ* from −1 to 1 is used to describe the chirality of the object^23^. Theoretical analysis shows that both the Poynting vector (**P**) and spin angular momentum (SAM) contribute to the optical lateral force on a dipole chiral particle^20,22^, i.e.,

1 $$F_{{\mathrm{lateral}}} = F_{{\mathrm{Poynting}}} + F_{{\mathrm{SAM}}} = \frac{{\sigma \left\langle {\mathbf{S}} \right\rangle }}{c} + \omega \gamma _e\left\langle {{\mathbf{L}}_e} \right\rangle$$

where $< {\mathbf{S}} > = 1/2{\Re} [{\mathbf{E}} \times {\mathbf{H}}^ \ast ]$ and $\left\langle {{\mathbf{L}}_e} \right\rangle$ are the time-averaged Poynting vector and electrical spin density, respectively. *σ* is the cross-section in vacuum. The lateral force resulting from SAM is usually one order of magnitude smaller than that from the Poynting vector^22^. Therefore, plotting the Poynting vector surrounding the chiral particle is an intuitive way to elucidate the optical forces. According to Minkowski’s approach^36^, the optical force increases *n* times in a dielectric medium (*n* is the refractive index of the medium) due to the momentum transfer; thus, the medium effect for a liquid with a higher refractive index is more prominent^37–40^.

A microscopic image of cholesteric polymerized microparticles between crossed polarizers is shown in Fig. 1b, where the light pattern (Maltese cross) on the particles comes from the supramolecular spherulitic arrangement, as sketched in the inset^41^. After UV exposure of the emulsion, the polymerized particles preserve both the spherical shape and internal supramolecular arrangement of the precursor cholesteric droplets, offering several advantages (compared to liquid crystal droplets) for optical manipulation experiments where stability of the shape and the internal configuration is required. Figure 1c, d shows SEM and TEM images of the particles, respectively. The TEM investigations manifest the self-organization in a radial configuration for our material at *R/p* ≥ 1.5, where *R* and *p* are the radius and pitch of the particles, respectively. Based on the above features, the polymeric microparticles can exhibit chirality at both the molecular (chiral additive molecules) and supramolecular levels, which offers a perfect paradigm for the experimental sorting of chiral particles in the Mie regime. The cholesteric particles immersed half in water and half in air are assumed to be lossless spheres with the real part of the refractive index equal to ~1.5 at 532 nm and a chirality *κ* of +0.4. Unlike the optical lateral force on dipole chiral particles (*R* ≤ 100 nm)^23^, whose sign depends only on the chirality of the particle, our results show that the sign of the optical lateral force on the micro-chiral particles (*R* ≥ 300 nm) could directly depend on the size (Fig. 1e, f) and chirality (Fig. 1g, h). The force map as a function of particle radius in Fig. 1e shows that the sign of the lateral force can be reversed by changing the particle size and the incident angle when *R* is on the order of the wavelength (Mie regime) for a fixed chirality *κ* = +0.4 and an s-polarized beam. The variation in the lateral force with the size and incident angle under the illumination of a p-polarized beam is shown in Fig. 1f. It is noted that the sign of the lateral force could be reversed under different polarizations of light at certain incident angles. For instance, the signs of the lateral forces on different-sized particles are opposite for s- and p-polarized beams when *θ* = 45°. This effect is also observed in the experiment. The lateral force could also be a function of *κ* for a fixed radius (*R* = 500 nm), as shown in Fig. 1g, h. For the s-polarized light, most lateral forces remain negative over a large range of kappa (*κ* < 0.5), while the forces are positive for p-polarized light over the same range.

### Analysis of the optical lateral force

**Fig. 2:**
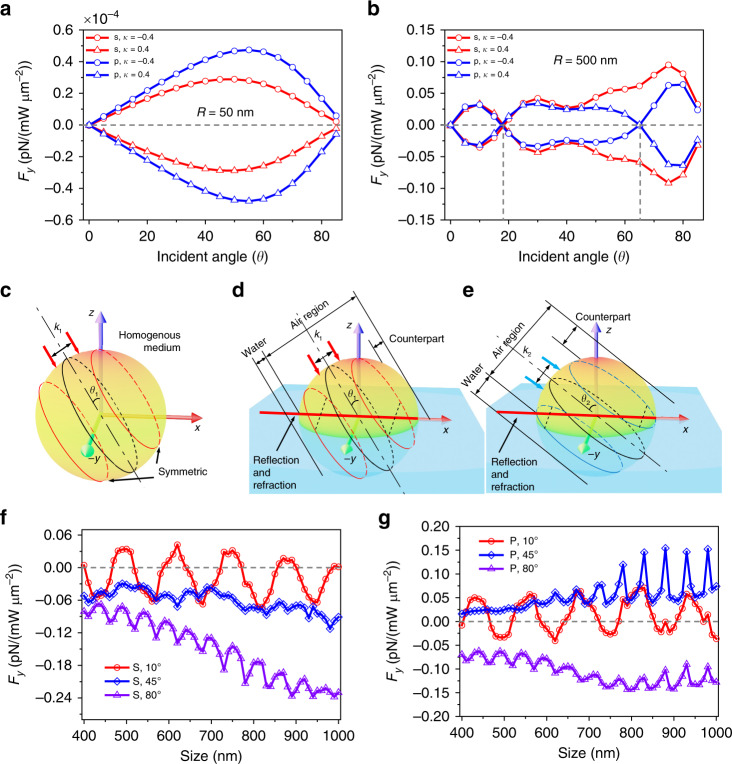
Reversible optical lateral force on Mie chiral particles induced by the effect of the incident angle and particle size. **a** Over the whole range of incident angles and for both s- and p-polarization, the 100-nm dipole chiral particle experiences positive and negative optical lateral forces when *κ* = −0.4 and *κ* = +0.4, respectively. **b** The sign of the optical lateral force reverses under different angles for the 1-μm chiral particle. **c**–**e** Sketch of the incident angle-induced optical lateral force. **f** The optical lateral force reverses sign when the incident angle is small (*θ* = 10°) even for large particles (*R* = 800‒1000 nm). **g** The sign of the lateral force at 45° is also different from that at 80° for large particles under the p-polarized beam

Previous theoretical predictions focused on chiral particles located either above or below the interface. The dipolar approximation, commonly used in the theoretical modeling of optical forces on chiral particles, indicates that the sign of the lateral force depends only on the sign of the chirality (kappa *κ*)^9,21–23^. For example, theoretical analysis^20–23^ shows that dipolar chiral particles with different chiralities *κ* > 0 and *κ* < 0 experience optical lateral forces to the left (*F*_*y*_ < 0) and right (*F*_*y*_ > 0), respectively. The sign is not affected by a change in the incident angle of light. Our simulations show that this is also true even if the dipolar particle (*R* = 50 nm) is located at the interface (e.g., half in air (*z* > 0) and half in water (*z* < 0)), as shown in Fig. 2a, where we plot the simulated lateral force versus the incident angle for isotropic chiral spheres with *κ* = +0.4 (triangles) and *κ* = −0.4 (circles). For both s- and p-polarized beams, the lateral forces are always negative for *κ* > 0 (positive for *κ* < 0) at any incident angle.

However, we found unexpected behavior for chiral particles with radius *R* = 500 nm, as shown in Fig. 2b. The force reverses sign with increasing incident angle at *θ* ≈ 18° for both s- and p-polarized beams. The sign reserves again at *θ* ≈ 66° for the p-polarization. Intuitively, we attribute this angle-induced effect to two reasons. Let us divide the plane wave into two regions separated by the axis *k*_1_ as shown in Fig. 2c–e. Two parallel light beams from different areas are shined on the boundary of the particle in the incident plane with identical distance to axis *k*_1_. Consider their scattering fields in two planes (marked as two red circles in Fig. 2c) parallel to *k*_1_. When the medium around the sphere is homogenous, the interaction of the two light beams inside the particle will result in a net zero force in the *y* direction because of symmetry, as shown in Fig. 2c. However, due to the interface, the portions of air and water in the two planes are different, resulting in different diffraction and momentum exchange at the boundary, which eventually generates a lateral force. The other reason is that the reflection from the interface induces additional light rays on the sphere. The different portions of the refraction area cause a change in the light path to the sphere. The reflection and refraction together contribute to the emergence of the lateral force. When the incident angle changes, the portions of air and water in the relevant planes (blue circles) in Fig. 2e will be different from that in Fig. 2d, resulting in different lateral forces. In addition, different incident angles have different ranges of the water region, where the reflection and refraction are different. We can also comprehend the origin of the optical lateral force on chiral particles by considering the linearly polarized beam as two circularly polarized beams with different handedness (see discussion below).

Plots of the lateral force on larger chiral particles (600 nm < *R* ≤ 1000 nm) are shown in Fig. 2f, g. Small incident angles (e.g., 10°) can easily induce a reversal of the lateral force. This is because when the incident angle is small, the energy is focused near the *z*-axis, where the size effect is more significant. A small change in size can extraordinarily affect the curvature of the particle boundary near the axis. This is very similar to the linear momentum transfer in the incident plane^42,43^. The optical lateral force can also have opposite signs at medium (*θ* = 45°) and large (*θ* = 80°) incident angles for a p-polarized beam, as shown in Fig. 2g. Meanwhile, the oscillations of the curves in Fig. 2f, g result from the size effect of Mie particles^44^. The lateral forces on multilayer particles are plotted in Supplementary Fig. S2, which shows that the force difference between inhomogeneous and homogenous chiral microparticles is not prominent. The force difference for small inhomogeneous particles (*R* = 250 nm) is negligible because of the weak momentum transfer when the particle size is less than the wavelength^23,37,43^. The lateral force and force difference become larger with increasing particle size. The force difference is more prominent at a larger incident angle, which can be explained by the sketch in Supplementary Fig. S3. In practice, the synthetic chiral particles tend to retain good performance in terms of chirality^35^. Moreover, the inhomogeneous effect can be eliminated by choosing a proper angle (e.g., 45°).

### Lateral momentum transfer on Mie chiral particles

**Fig. 3:**
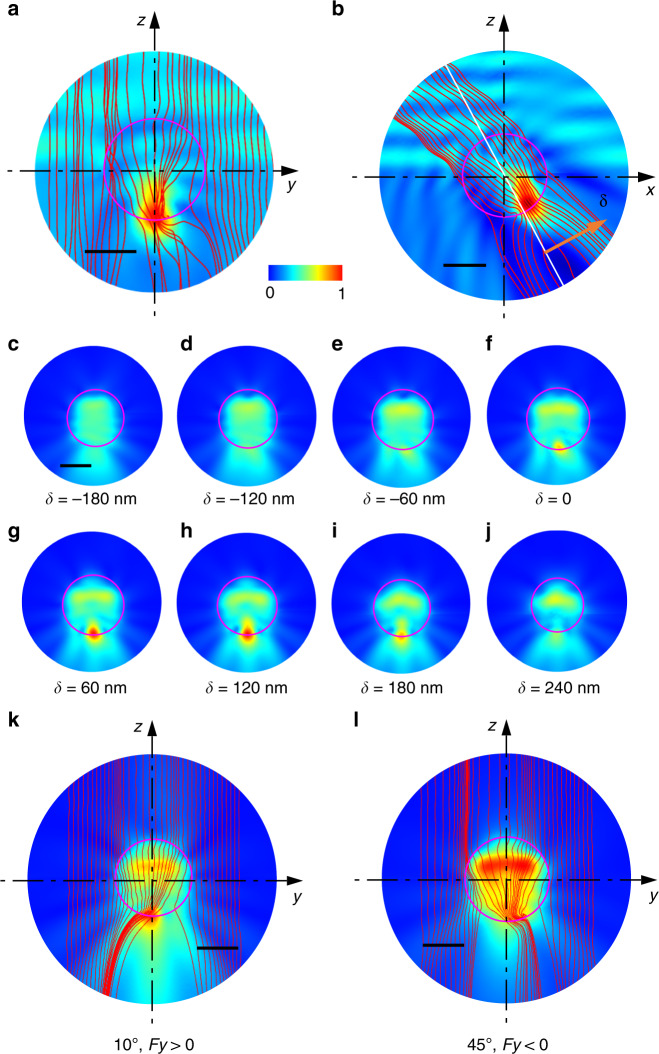
Simulation of the optical field and Poynting vector for the optical lateral force. **a** *y*–*z* projection of the electric field and 3D Poynting vector of a chiral particle (*κ* = +0.4) under the illumination of an s-polarized beam with an incident angle of 45°. **b** Normalized electric field in the *x*–*z* plane. **c**–**j** Plot of the scattering fields in different planes perpendicular to the direction of δ from −180 to 240 nm. The direction of δ is perpendicular to the white dashed line with d*z*/d*x* = −2 in the *x*–*z* plane. **c**–**j** share the same scale bar of 500 nm. **k**, **l** Electric field and 2D Poynting vector in the *y*–*z* plane for *θ* of 10° (**k**) and 45° (**l**). The scale bars in **k**, **l** equal 500 nm. All subfigures share the same color bar

To comprehend the optical lateral force, we plot the *y*–*z* view of the 3D distribution of the time-averaged Poynting vector surrounding a chiral particle with a radius of 500 nm, as shown in Fig. 3a. The particle with chirality *κ* = +0.4 is placed at an air–water interface (half in air (*z* > 0) and half in water (*z* < 0)) and illuminated by an s-polarized plane wave with an incident angle of 45°. The helix structure of the chiral particle causes the energy flow to spiral and scatter away from the incident plane (*x*–*z*) to the lateral plane (*y*–*z*). The energy flow then passes through the surface of the chiral particle and goes into the air and water regions, causing momentum exchange and generating the optical lateral force. The energy flux has distinct asymmetry and higher density in the water region, especially near the particle boundary. It is worth noting that the lateral force *F*_lateral_ should be multiplied by the refractive index *n*, which is the refractive index of water (1.33) or air (~1), based on the Minkowski stress tensor. Therefore, the net force in the *y* direction is dominantly contributed by the energy scattered from the particle to water. The normalized electric field is denser in the +*y* direction, as shown in the background of Fig. 3a. At the same time, most Poynting vectors point in the +*y* direction from the particle to water, resulting in a negative force *F*_*y*_. Since the light is obliquely incident, the normalized electric field is focused in the water after passing through the particle, as shown in Fig. 3b. For chiral particles, our results indicate that the lateral forces arise from a complex interplay between the “out-of-plane” light scattering from the chiral particle to air and water and the abovementioned “in-plane” momentum exchange.

To obtain a comprehensive view of the energy scattering from the chiral particle, we show slices of the scattering field along the direction of δ in Fig. 3c–j. The energy scattering has a bias in the +*y* direction when δ is from −180 to +120 nm. Only slightly more energy is scattered in the –*y* direction when δ ranges from +180 to +240 nm. As the scattering field is densest and shows a clear bias toward the +*y* direction in the plane from δ = +60 and +120 nm, the net energy is scattered in the +*y* direction, resulting in a negative optical lateral force. The chiral particle with *κ* = +0.4 experiences a positive lateral force when *θ* < 18°, which can be explained by the plot of the electric field and Poynting vector in the *y*–*z* plane at *x* = 200 nm, as shown in Fig. 3k. It shows a distinct bias of energy scattering toward the –*y* direction when *θ* = 10°. The energy scattering direction reverses when *θ* = 45° (*x* = 0), as shown in Fig. 3l. Detailed simulations of the lateral momentum transfer when *θ* = 0° and 45° are shown in Supplementary Figs. S4 and S5, respectively. It is noted that because *F*_*y*_ is much smaller when *θ* = 10° than when *θ* = 45°, the momentum transfer has a different bias in different layers. It is safe to deduce the optical force using the overall 3D Poynting vector in Supplementary Fig. S4a or using the numerical results in Fig. 1e. It is unambiguous that the momentum has a distinct bias towards the +*y* direction in most of the layers for *θ* = 45°, resulting in *F*_*y*_ < 0. Figure 3k, l is chosen to represent the net momentum transfer under different angles. More simulations of the lateral momentum transfer under different incident angles, polarizations, chiralities and sizes can be found in Supplementary Figs. S6–S9.

### Experimental setup and sample characterization

**Fig. 4:**
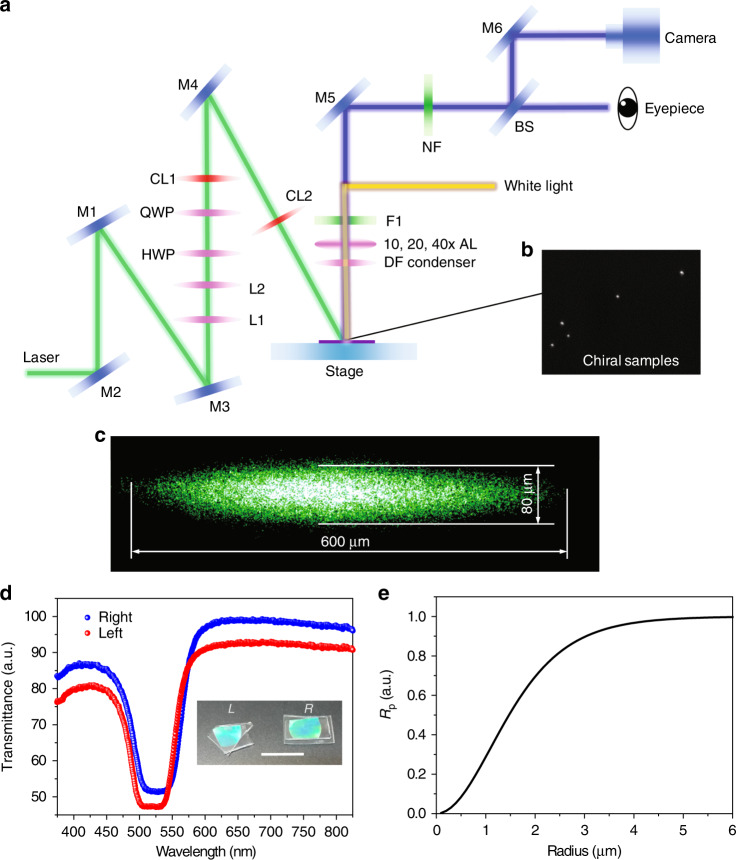
Experimental setup and sample characterization. **a** Experimental setup. M mirror, L1 lens 1 (15 mm), L2 lens 2 (200 mm), HWP half-wave plate, QWP quarter-wave plate, C1 cylindrical lens 1 (300 mm), C2 cylindrical lens 2 (100 mm), AL air lens, BS beam splitter, DF dark field, NF notch filter. **b** Dark-field image of chiral particles. **c** Observed beam profile for an incident angle of 45°. The profile was taken by placing thin tape over the surface of the square well in the chip. **d** Transmission spectra of polymerized cholesteric films of left- and right-handed mixtures, displaying superimposed reflection bands that reveal the same pitch of the self-organized helicoidal structures. The scale bar equals 2 cm. **e** *R*_*p*_ versus radius of the particle evaluated for chiral particles with *p* ≈ 330 nm

To observe the lateral movement of Mie chiral particles, a line-shaped laser spot for creating a line trap was introduced into a microscope stage where an optofluidic chip was placed, as shown in Fig. 4a–c. The dimensions of the laser spot were kept at 80 × 600 μm^2^, controlled by two cylindrical lenses, as shown in Fig. 4c. The 80-µm width is used to generate an optical gradient force to confine microparticles inside the line trap. The 600-µm length mitigates the influence of the optical gradient force on the lateral force. The optical gradient force in the lateral (*y*-) direction is negligible compared to the optical lateral force (see Supplementary Figs. S10 and S11 for detailed simulations). Chiral particles were synthesized with resonance at 532 nm, as shown in Fig. 4d. The polymeric microparticles exhibit chirality at both the molecular (chiral additive molecules) and supramolecular levels. The chiral supramolecular contribution gives rise to a Bragg-reflection phenomenon for circularly polarized light with the same handedness as the particle chirality and wavelength in a proper range (*n*_⊥_*p* < *λ* < $n_{II}p$, where *n*_⊥_ and $n_{II}$ are the refractive indices perpendicular and parallel to the molecular direction, respectively; *p* is the pitch of the helicoidal supramolecular organization). Omnidirectional reflection occurs based on the supramolecular radial configuration of the helices, while the handedness of the reflected circularly polarized light (CPL) is preserved, acting as a chiral mirror. Depending on the particle chirality, the CPL with opposite handedness propagates with a constant refractive index $\bar n = \frac{{n_{II} + n_ \bot }}{2} = 1.5$. The antiparallel reflectance value *R*_*ap*_ can be evaluated as the average over the two orthogonal polarization directions with respect to the incidence plane, which can be expressed using the equation $R_{ap} = \frac{1}{2}\left( {\frac{{{\mathrm{sin}}^2\left( {\theta} \,-\, {\beta } \right)}}{{{\mathrm{sin}}^2\left( {\theta} \,+\, {\beta } \right)}} + \frac{{{\mathrm{tan}}^2\left( {\theta} \,-\, {\beta } \right)}}{{{\mathrm{tan}}^2\left( {\theta} \,+\, {\beta } \right)}}} \right)$, where *θ* is the incidence angle at the surface of the sphere and *β* is the refraction angle. In contrast, the CP light with the same handedness as the helix handedness and wavelength within the selective reflection band can be strongly reflected, and the reflectance *R*_*p*_ can be evaluated from $R_p = \left| {\tan h\left( {\frac{{\sqrt 2 \pi \left( {n_{II}^2 - n_ \bot ^2} \right)R}}{{3\lambda \sqrt {\left( {n_{II}^2 + n_ \bot ^2} \right)} }}} \right)} \right|^2$. Finally, the value of particle reflectance *R*_s_ depending on the light polarization, the particle size and the light wavelength can be expressed as^45,46^

2 $$R_s = R_p\left( {\frac{{1 + \sin 2\phi }}{2}} \right) + R_{ap}$$

where *ϕ* is the ellipticity angle. *R*_*ap*_, which is related to the refractive index difference at the air–particle interface, has a value of ~0.05. Therefore, *R*_*p*_ is only related to the radius of the particle for the present case, as plotted in Fig. 4e.

For particles with *R* ≥ 6 μm, *R*_*p*_ can reach a value of 1, i.e., the CP parallel component is completely reflected. Since the particles exploited in the experiment have a radius from 0.5–1 μm, *R*_*p*_ ranges from 0.08 to 0.28, as shown in Fig. 4e. The expected *R*_s_ at the air–particle interface is in the range of 9‒19%. Because the absorption of the polymer as well as the circular dichroism is very low in the visible range, the transmittance *T* ≈ 1 − *R*_s_. Based on this assumption, we can introduce and evaluate a “structural dichroism” $D = \frac{{T_ + - T_ - }}{{T_ + + T_ - }}$, where *T*_+/−_ are the transmittances for left/right CP light. *D* ranges from 0 to (+/−) 1 for (left/right) chiral particles with *R* ≤ 6 μm and is (+/−) 1 for (left/right) chiral particles with *R* ≥ 6 μm. As discussed above, the handedness of CP beams affects the reflectivity of chiral particles.

When this effect is strong (*R*_*p*_ = 1), the radiation pressure dominates, while for *R*_*p*_ < 0.3, the radiation pressure is reduced and the effect of the lateral force (lateral scattering) on microparticles at the interface occurs. We can also expect different scattering efficiencies in the lateral direction for different CP beams. The optical lateral force on the chiral microparticles can be comprehended by dividing the linearly polarized beam into two CP beams with different helicities.

### Experimental demonstration of the bidirectional sorting of Mie chiral particles

**Fig. 5:**
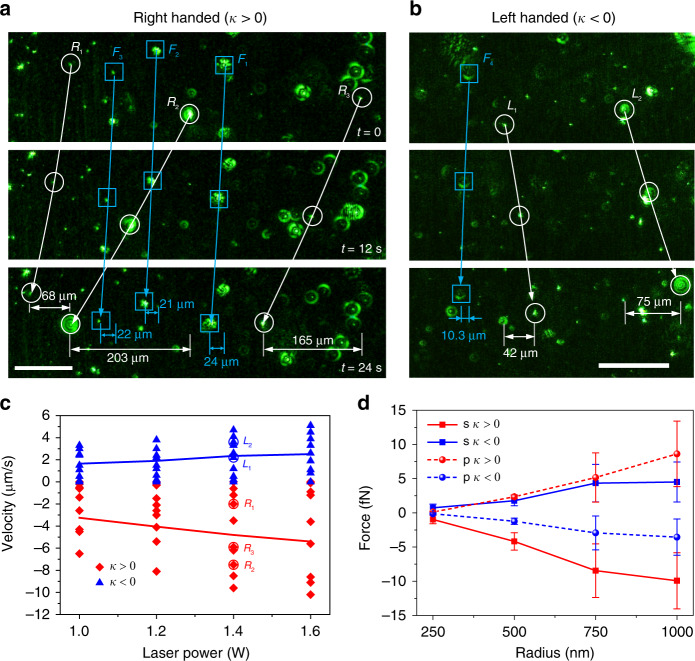
Experimental demonstration of sorting of chiral particles in a line trap. **a**, **b** Chiral particles with *κ* > 0 (**a**) and *κ* < 0 (**b**) experience lateral forces to the left (*R*_1_, *R*_2_, and *R*_3_) and right (*L*_1_ and *L*_2_), respectively. Weak coupling particles (labeled *F*_1_, *F*_2_, and *F*_3_) could be affected by the background flow and move with almost the same velocities. Different particles have different velocities due to the different sizes and slightly different chiralities. The laser power used was 1.4 W. The scale bars in **a**, **b** equal 100 µm. **c** Measured velocities of particles with different chiralities under different laser powers. **d** Measured maximum optical lateral force in each video sequence with varying particle size and polarization of light. The lateral forces have opposite signs under s- and p-polarized beams when the chirality is the same

Bidirectional sorting of polymeric particles performed at room temperature (20 °C) is shown in Fig. 5a–d. The particles were initially passed through a mechanical filter with 2-µm pores to eliminate particles larger than 2 µm. To avoid or mitigate the complex dependence of lateral forces on the size and chirality, we used s- and p-polarized beams with an incident angle *θ* = 45° in the experiment according to the simulation results in Figs. 1 and 2. Particles were then freely floated at the air–water interface. Due to the preparation process, some particles with small sizes or slightly different pitches presented weak chirality coupling, which served as references for the lateral movement. Because of the particularities of the experiment and the small particle size, the scattered light of chiral microparticles was used to observe the lateral displacements (see Supplementary Fig. S12). When illuminated with the s-polarized laser beam, the particles with weak chirality coupling were stably trapped inside the line trap, as shown in Fig. 5a, b. Three right-handed microparticles (*κ* > 0, marked with white circles) experienced an optical lateral force in the –*y* direction, as shown in Fig. 5a. They had different velocities because of the different sizes and chirality couplings. The maximum velocity of the three particles was ‒8.5 μm/s. The reference particle (marked with white squares) with negligible optical lateral force had an only 21-μm lateral displacement in 24 s, resulting in a velocity of −0.9 μm/s. This movement was caused by the heating-induced vibration of the background flow. Since the polymerized chiral particle and water had negligible absorption of 532 nm light, the velocities of the background flow induced by the heating were normally less than 1 μm/s, which were much smaller than the velocities induced by the lateral forces. Meanwhile, this vibration could be easily characterized by observing particles with the same slow velocity (e.g., *F*_1_, *F*_2_ and *F*_3_ in Fig. 5a) and could be easily eliminated by subtracting this velocity from the overall velocities of chiral microparticles (see Supplementary Fig. S13 for more results). The background particle movement could result from the heating-induced thermophoretic force^47,48^, which can be estimated using $F_t = - 9\pi R\eta ^2\Delta T/\left( {2 + C_m/C_p} \right)/(\rho T)$^49^, where *R* and *C*_*p*_ are the radius and thermal conductivity of the particle, respectively. *η*, *C*_*m*_, *ρ*, *T*, and ∆*T* are the viscosity, thermal conductivity, density, temperature, and temperature gradient of the medium, respectively. Since particles were placed half in air and half in water, the optical forces could be deduced from the velocities of particles and expressed as *F*_drag_ = 0.5 × 6 *πηRv*, where *η* is the viscosity of the liquid and *R* and *v* are the radius and velocity of the particle, respectively. Substituting the velocity of 1 µm/s into the equation *F*_t_ = *F*_drag_, we obtained the equivalent temperature gradient of ~0.2 °C/mm, which could be reached when a laser beam is focused on glass or into water with salt or other chemicals^47,48^.

Two left-handed particles (*κ* < 0, marked with white circles) experienced optical forces in the +*y* direction, as shown in Fig. 5b. The maximum velocity of the two particles was +3.1 μm/s. The background flow velocity was −0.4 μm/s. The velocities of particles with different handedness under different laser powers are shown in Fig. 5c. When illuminated with an s-polarized beam, particles with *κ* > 0 and *κ* < 0 experienced optical forces in the −*y* and +*y* directions, respectively. The velocities linearly increased with laser power, showing good feasibility of our method for sorting particles with different chiralities. The averaged velocities of particles with *κ* > 0 were approximately twice those of particles with *κ* < 0. The velocities were obtained from the maximum velocities for different sizes in each video. The absolute value of the lateral force increased almost linearly with particle size for both s- and p-polarizations when the radius increased from 250 to 1000 nm, as shown in Fig. 5d. Interestingly, the directions of the lateral forces for the p-polarization were opposite to those for the s-polarization, in accordance with the simulation results in Fig. 2f, g. The absolute values of the lateral forces for small particles (*R* = 250 nm) under the illumination of the p-polarized beam were much smaller than those under the illumination of the s-polarized beam. However, the lateral forces did not differ greatly for larger particles (*R* > 250 nm). This effect also coincides with the simulation results. Therefore, the s-polarized beam was a better option for bidirectional sorting of Mie chiral particles than the p-polarized beam.

## Discussion

One may have the following question: are there any high-order multipoles in the Mie chiral particles? Recently, broad interest has emerged in the study of intriguing high-order multipoles in dielectric elements, including the multipoles and bound states in the continuum (BIC) in nanocylinders^50,51^, as well as the multipole resonance enhanced second harmonic generation (SHG) in AlGaAs (aluminium gallium arsenide)^52^. The existence of electric and magnetic modes enhances the scattering cross sections and optical forces. We could also expect these high-order modes in chiral particles and enhanced optical forces (both radiation and lateral). However, the appearance of these high-order multipoles requires some criteria to be met, e.g., a high refractive index (normally *RI* > 3), a small size (normally < wavelength/2), and a specific structure (e.g., specific length/radius ratio in cylinders). Since our chiral particles have a low refractive index (~1.5) and a relatively large size (~wavelength), high-order multipoles are unlikely to occur. This can also be concluded from the force maps in Fig. 1e–h, as the distribution of optical force does not have any abrupt change coming from multipoles.

In summary, we reveal an unexpected behavior of chirality-dependent lateral forces when chiral microparticles in the Mie regime are located at the interface between air and water. Our numerical simulations show that the sign of the optical lateral force depends not only on the chirality, as expected from the dipole approximation in previous papers, but also strongly on the incident angle, beam polarization, and particle size. The sign reversal of the chirality-dependent lateral force can be regarded as a chiral analogue of “negative” forces or “left-handed” torques. In practice, by choosing s- and p-polarized beams with an incident angle of 45°, for the first time, we demonstrate sorting of Mie cholesteric polymeric microparticles using an optical lateral force. Particles with left and right chirality experience optical lateral forces with opposite directions. Particles with the same chirality experience opposite optical lateral forces under s- and p-polarized beams when *θ* = 45°. Our studies on Mie chiral microparticles complete the understanding of the recent theoretically proposed extraordinary optical lateral force from the aspect of momentum transfer and open up new avenues for probing and sorting of micro-objects with different chiralities.

## Materials and methods

### Sample preparation and characterization

Polymerized liquid crystal microparticles were produced via UV irradiation of micron-sized droplet emulsions of photopolymerizable cholesteric liquid crystals in water. A nematic reactive mesogen, RMS03-001C (Merck KGaA, Germany), was used after solvent evaporation. The cholesteric phase was achieved by doping it with a chiral agent. The molar circular dichroism of *R*/S811 was measured in the blue–green region of the spectrum by exploiting a mixture of the chiral dopants in ethanol at a concentration of 1.4% by weight. The measured value of the molar circular dichroism for both chemical agents is ∆*ε* ≈ 1 cm^−1^.

To produce left- and right-handed microparticles, two different mixtures were prepared with a left-handed (ZLI-811 Merck KGaA, Germany) or a right-handed (ZLI-3786 Merck KGaA, Germany) chiral agent. The left- and right-handed chiral dopants lead to a left or right rotation of the nematic director, inducing a left-handed or right-handed supramolecular helicoidal structure, respectively. The chiral dopant concentration was fixed at 22.5 wt% for both mixtures to achieve helicoidal structures with a pitch of ~330 nm, which leads to enhanced coupling with the 532 nm laser beam. Among the different techniques used to manufacture cholesteric droplets, including emulsification and microfluidics approaches, the only feasible method here is emulsification due to the high viscosity of the reactive mesogen. The cholesteric microdroplets were obtained in aqueous emulsions by adding 0.5 wt% of the chiral mesogen mixture into ultrapure water (≥18.2 M_@25 °C, Synergy UV, Millipore), which produced a parallel (i.e., planar) molecular orientation at the interface. The blends were shaken at 20 Hz for 30 s at 90 °C in a glass vessel using a laboratory vortex mixer. Subsequently, polymerized chiral particles were obtained by exposing the emulsions to a 2 mW/cm^2^ UV lamp (*λ* = 365 nm, LV202-E, Mega Electronics) at room temperature for 6 h under nitrogen flux. The resulting chiral solid microparticles preserve both the spherical shape and internal supramolecular arrangement of the precursor liquid crystal droplets, allowing the experimental investigation of floating microparticles^35^. The optical microscope observations reveal that almost all the microparticles have a radial configuration of the helix axes of the particles, while a small pitch dispersion is displayed by the reflected color. The average refractive index of the polymeric chiral particles is 1.5 at 532 nm. The suspension was initially passed through a 2-µm mechanical filter to eliminate particles larger than 2 µm. Dynamic light-scattering (Zetasizer Nano ZS, Malvern) measurements were performed, and a polydispersity index PDI = 0.35 was measured. The transmission spectra of the left- and right-handed polymers are shown in Fig. 4d. Since the density of the microparticles is higher than that of DI water, we used saturated potassium chloride (KC1) deionized water (DI) water to float them on the surface. The refractive index of the saturated KC1 solution at 20° is ~1.336.

Due to the low absorption of the materials at the used wavelength, the value of the molecular circular dichroism is very small. However, at this wavelength, the circular dichroism stems from diffraction of light^46,53,54^. A Bragg-reflection phenomenon^46,53,54^ occurs for circularly polarized light with the same handedness as the material/particle chirality due to the supramolecular shell arrangement. Such “structural dichroism” can be evaluated by the difference between the transmission coefficients of the two circular polarizations, $D = \frac{{T_ + - T_ - }}{{T_ + + T_{ - l}}}$, where *T*_+/−_ are the transmittances for left/right CP light^46,53,54^. Accordingly, omnidirectional uniform reflectance occurs for particles with a radial configuration of the helical axes, as shown in Fig. 1d.

The structural dichroism *D* strongly depends on the *R/p* ratio^46,53,54^. For large particles ((*R/p*) > 12, see Fig. 4e), *D* is $\cong\!\pm\! 1$ depending on the particle chirality, and the optical force induced by radiation pressure dominates. Conversely, for small particles, the radiation pressure force is reduced, allowing other optomechanical phenomena to be observed^46,53,54^, as in the present case. Indeed, the value of *D* ranges from nearly 0 (for *R* < 500 nm) to 0.06 (for *R* ≈ 1000 nm).

Therefore, based on the above issues, polymeric microparticles with sizes <2 µm exhibit unique features that enable experimentally investigation of the lateral force and reliable fit of the approximation of spherical particles with uniform chirality adopted in the theoretical modeling. More images of the polymeric chiral microparticles can be found in Supplementary Fig. S14.

### SEM and TEM measurements

SEM (Quanta 400 FEG, FEI) analysis was carried out in low vacuum on fully polymerized microparticles after water evaporation. To perform TEM measurements, the polymeric microparticles were first embedded in an epoxy resin (Araldite, Fluka) and successively cut into ultrathin sections of ~100 nm by a diamond knife. The ultrathin sections were collected on copper grids and then examined with a Zeiss EM10 transmission electron microscope at an 80 kV acceleration voltage. The concentric ring structures observed in the TEM images in Fig. 1d correspond to the topography of the thin slices. These corrugations are due to the cutting process and occur due to a certain orientation of the molecular director **n** with respect to the cutting direction. Moreover, the equidistance between dark and bright concentric rings suggests that the investigated section was within an equatorial region of the particle.

### Chip fabrication and experimental setup

The optofluidic chip was made from polydimethylsiloxane (PDMS)^55^. A PDMS slice was first cut into a block (2 × 2 cm^2^). A square well (5 × 5 mm^2^) was drilled at the center of this block using a scalpel. Then, the PDMS block was bonded to a cover slide (0.17 mm) using plasma treatment^56^. The whole chip was placed onto the stage of an inverted optical microscope (TS 100 Eclipse, Nikon). It was then covered by a culture dish to prevent environmental disturbance from air flow. A c.w. laser (532 nm, Laser Quantum, mpc 6000; laser power, 2 W) was obliquely incident into the holes. The beam was focused into a line trap using a combination of two cylindrical lenses with focal lengths of 300 and 100 mm. The area of this line trap was kept at 80 × 600 μm^2^ to trap microparticles inside and minimize the lateral gradient force. The chiral microparticles at the air–water interface were imaged through a ×10 microscope objective (NA 0.25, Nikon) using a charge-coupled device camera (Photron Fastcam SA3) with a frame rate of 125 frames per second.

### Simulation details and constitutive relations of chiral particles

We simulated the Poynting vector and optical lateral force in COMSOL by applying the constitutive relations of a chiral particle, which can be expressed as

$${\mathbf{D}} = \varepsilon _r\varepsilon _0{\mathbf{E}} + i\kappa /c{\mathbf{H}}$$

$${\mathbf{B}} = - i\kappa /c{\mathbf{E}} + \mu _r\mu _0{\mathbf{H}}$$

where *ε*_*r*_ and *µ*_*r*_ are the relative permittivity and permeability of the chiral particle, respectively. The sign of kappa (*κ*) is positive, negative, and zero when the chiral particle is right-handed, left-handed, and nonchiral, respectively. The particle was placed at the interface of water (refractive index *n* = 1.33) and air (*n* = 1) under the illumination of a plane wave (wavelength *λ* = 532 nm). The simulation was conducted in a sphere with a diameter of 2 µm and a PML boundary condition. The maximum size of the mesh was set to *λ*/8/*n*, with *n* being the refractive index of the different media. The optical force was calculated using the Minkowski stress tensor written in COMSOL.

## Supplementary information

Supplementary Information for Chirality-assisted lateral momentum transfer for bidirectional enantioselective separation
