# Supplementary material for: Chirality-assisted lateral momentum transfer for bidirectional enantioselective separation
Source: Light Sci Appl. 2020 Apr 16;9:62. doi: 10.1038/s41377-020-0293-0 (PMC7160209; doi:10.1038/s41377-020-0293-0)
Supplement: Supplementary file 1 — Supplementary Information for Chirality-assisted lateral momentum transfer for bidirectional enantioselective separation [file 41377_2020_293_MOESM1_ESM.docx]

**Supplementary Information for**

**Chirality-assisted lateral momentum transfer for bidirectional enantioselective separation**

Yuzhi Shi^1, 2+^, Tongtong Zhu^3, 4, 5+^, Tianhang Zhang^3^, Alfredo Mazzulla^6^, Din Ping Tsai^7^, Weiqiang Ding^5^, Ai Qun Liu^2^, Gabriella Cipparrone^6,8^, Juan José Sáenz^9^ and Cheng-Wei Qiu^3*^

*^1^ School of Mechanical Engineering, Xi’an Jiaotong University, Xi’an 710049, China*

*^2^ School of Electrical and Electronic Engineering, Nanyang Technological University, Singapore 639798*

*^3^ Department of Electrical and Computer Engineering, National University of Singapore, Singapore 117583*

*^4^ School of Optoelectronic Engineering and Instrumentation Science, Dalian University of Technology, Dalian, 116024, China*

*^5^ School of Physics, Harbin Institute of Technology, Harbin, 150001, China*

*^6^ CNR-NANOTEC, LiCryL and Centre of Excellence CEMIF. CAL, Ponte P. Bucci, Cubo 33B, 87036 Rende (CS), Italy*

*^7^* *Department of Electronic and Information Engineering, The Hong Kong Polytechnic University, Hung Hom, Kowloon, Hong Kong, China*

*^8^ Department of Physics, University of Calabria, Ponte P. Bucci, Cubo 33B, 87036 Rende (CS), Italy*

*^9^ Donostia International Physics Center, 20018 Donostia-San Sebastián, Spain*

^+^*These authors contributed equally to the paper*

**Correspondence: Cheng-Wei Qiu (chengwei.qiu@nus.edu.sg)*

**Supplementary Note**

**Structural property of Mie chiral particles**

Chiral polymeric particles with spherical shape have been exploited to perform experimental investigation on the lateral force experienced by the particles floating at the air-water interface. The particles are created by photo-polymerization of chiral nematic liquid crystal droplets based on a reactive mesogen doped with chiral agents (chiral dopants, R811/S811). Spherical droplets are generated in pure water due to the material hydrophobicity, while the liquid crystal molecules align parallel at the water interface. Moreover, the internal configuration of the droplets self-organizes in supramolecular helicoidal structures of the mesogenic units having the same handedness of the chiral dopant. The spatial periodicity (pitch, *p*) depends on the percentage of the chiral agents added to the mesogenic blend. The arrangement of the helicoidal structures confined in micro-spheres strongly depends on the droplet radius *R* with respect to the pitch *p* of the helicity and the mesogen molecules orientation at the water interface, as well as physical parameters of the material (elastic constants, viscosity, etc). In the high chirality regime (*R/p* >> 1), the more favorite configuration is the radial one, where the helical axes are radial and an onion-shell configuration of the cholesteric layers. When the order of magnitude of *R/p* is in the range 0.1‒1, the droplets can self-organize in different supramolecular structures, including onion-shell, spiral or twisted bipolar configurations, which strongly depend on the material’s physical parameters.

**Reasons for choosing 45° as the incident angle in the experiment.**

i) From **Figs. 1e** and **1f**, the sign of the chirality-dependent lateral forces does not change with particle size (within the radius range of 400 nm to 1000 nm (see **Figs. 2f** and **2e**)) for both s-and p-polarized beams when *θ* = 45°.

ii) From **Fig. 1g** and **1h**, the sign of optical lateral force remains constant for both s-and p-polarized beams when *κ* is from 0 to 0.5 and the incident angle is 45°.

iii) From **Figs. 1e**-**h**, small incident angles can easily induce the reverse of optical lateral force with different chirality and size. Therefore, the small incident angle is not appropriate for the sorting. Meanwhile, the laser beam could be blocked by the objective lens at small incident angles. The beam will expand at large incident angles.


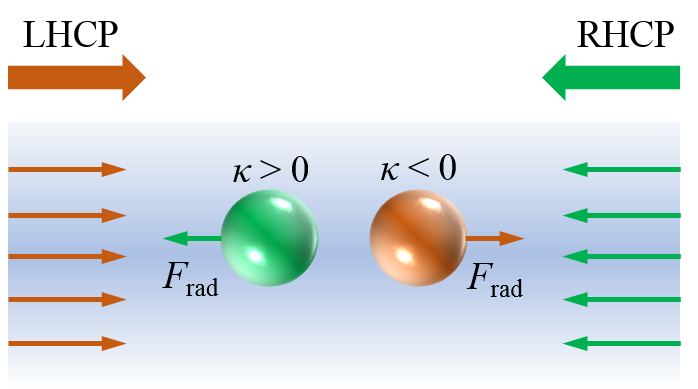

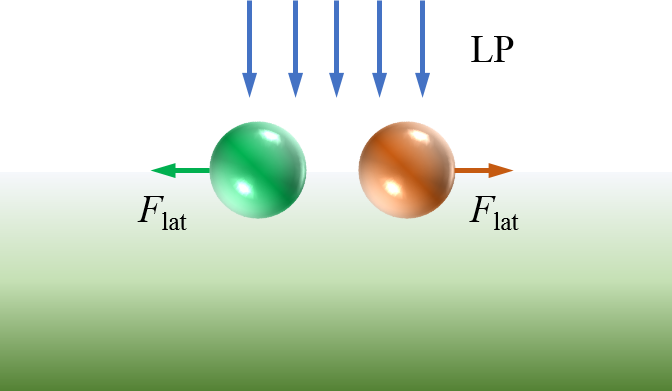


(a)

(b)

Fig. S1. Comparison of optical radiation force and lateral force on chiral particles. (a) Optical enantioselective sorting using two counterpropagating beams with different helicities. LHCP: Left-handed circularly polarization. RHCP: Right-handed circularly polarization. *F*_rad_: Optical radiation force. (b) Optical Enantioselective Sorting using a single linearly polarized beam. LP: Linearly polarization. *F*_lat_: Optical lateral force.

(c)

(d)

(a)


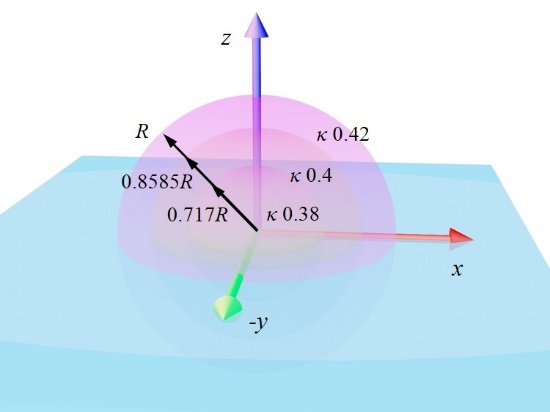

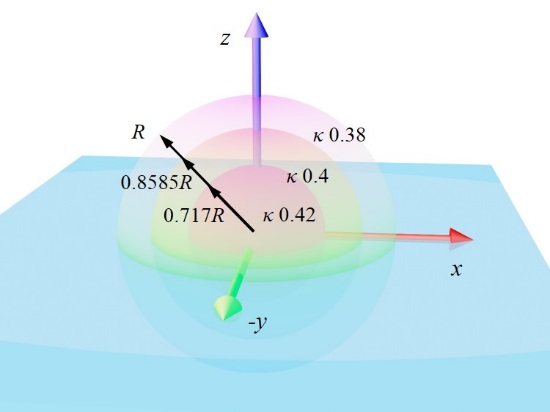


(b)

Fig. S2. Reversible optical lateral force on the inhomogeneous Mie chiral particles. (a) Illustration of inhomogeneous distribution of chirality in the chiral particle. We investigate two cases with different *κ* (+0.38, +0.4, +0.42) and (+0.42, +0.4, +0.38) from inner to outer space of the particle in (a) and (b), respectively. The dimensions of two boundaries are set to *R*_1_ = 0.717*R* and *R*_2_ = 0.8585*R* in order or keep the equivalent kappa to +0.4, e.g., $0.38\pi R_{1}^{3}+0.4(R_{2}^{3}-R_{1}^{3})+0.42(R^{3}-R_{2}^{3})=0.4R^{3}$. Optical lateral force on the inhomogeneous particles under the illumination of (c) s-polarized and (d) p-polarized beams. The inner +0.38 and outer +0.42 structure induces smaller optical lateral force comparing to the +0.4 structure. While, the lateral force from inner +0.42, outer +0.38 is larger than the +0.4 structure.

(a)

(b)


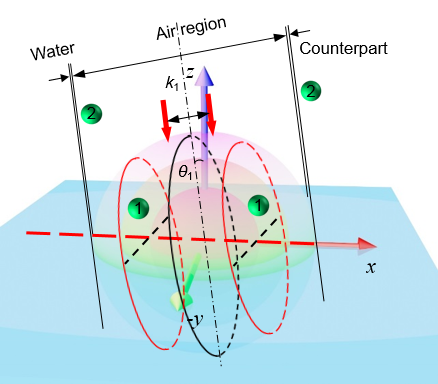

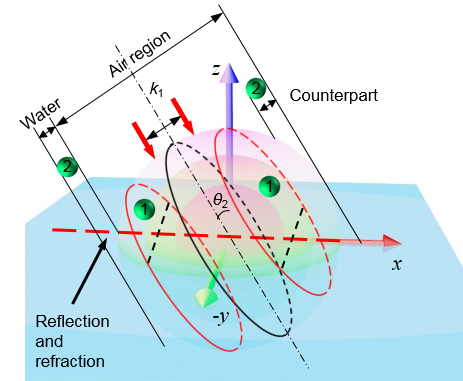


Fig. S3. Sketch of incident angle induced different lateral force on the three-layer chiral particles. The difference of air and water portions in red circles (labelled as factor “1”) in (a) is not as prominent as that in (b), meaning the force difference is smaller in (a) comparing to that in (b). Meanwhile, the smaller incident angle in (a) generates much smaller counterpart of air and water (labelled as factor “2”) than (b), being the second factor that the force difference at small incident angles is much smaller than large incident angles in Figs. 1(e)-(h).


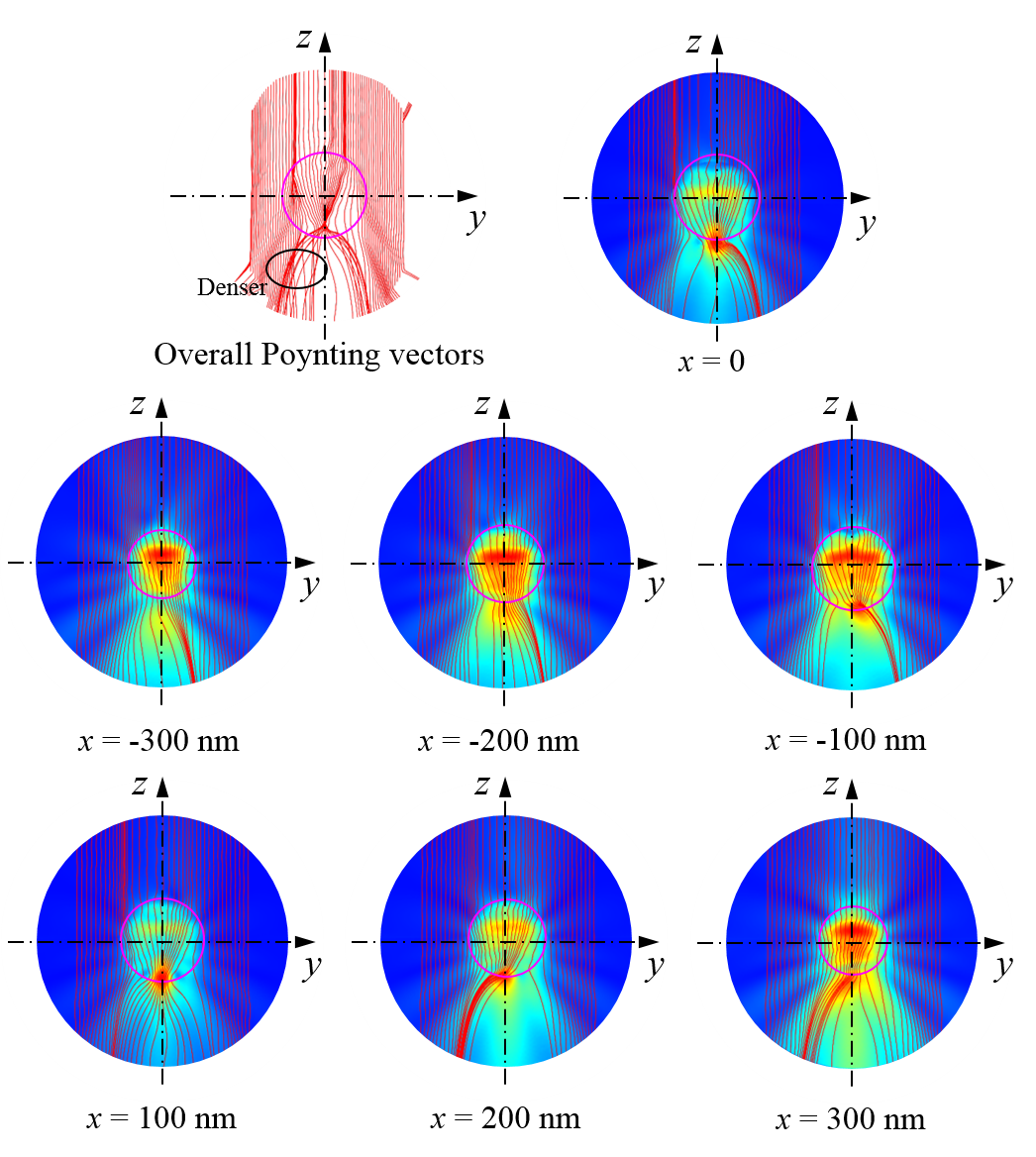


(a)

(c)

(b)

Fig. S4. Analysis of the momentum transfer on the chiral particle when *θ* = 10°, *κ* = +0.4 and the beam is s-polarization. (a) *y*-*z* projection of the 3D Poynting vector of a chiral particle. The Poynting vectors are denser in the -*y* direction, resulting in *F_y_* > 0. (b) The scattering electric field and 2D Poynting vector in the *y*-*z* plane when (b) *x* = 0 and (c) other layers in the *x*-direction.

It is noted that the momentum has different bias in different layers when *θ* = 10° because *F_y_* is much smaller than *θ* = 45°. It is safe to deduce the optical force using the overall 3D Poynting vector in Fig. S4(a) or the numerical results in Fig. 1(e).


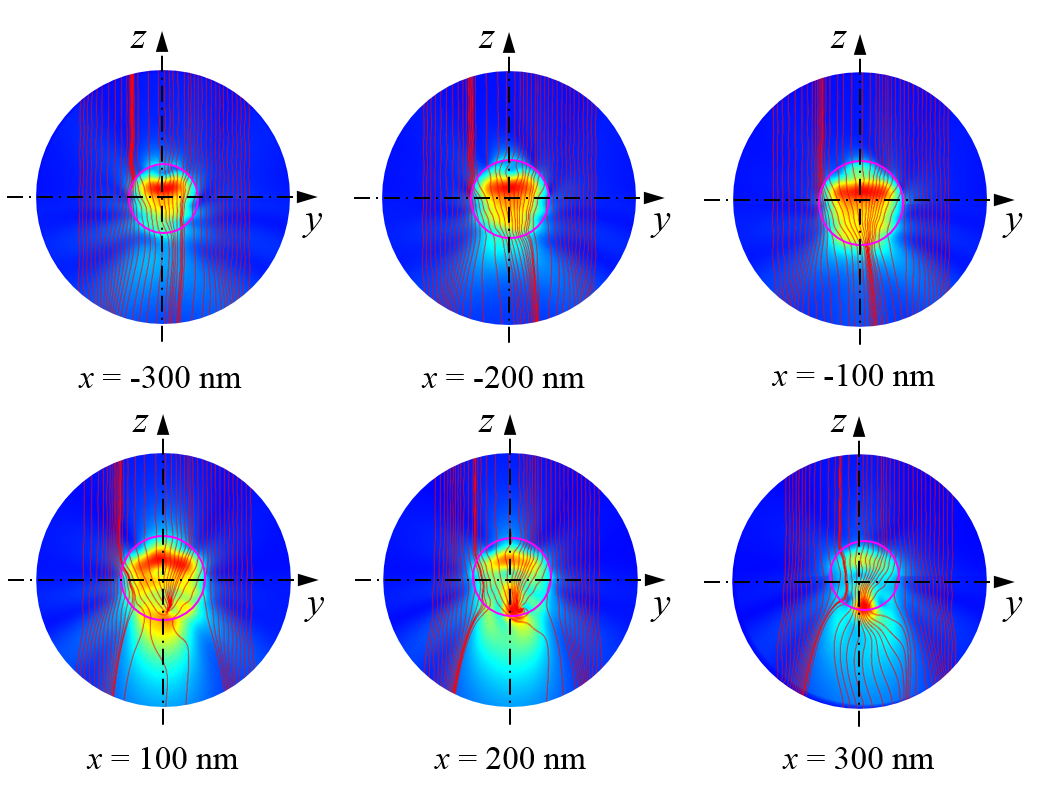


Fig. S5. The scattering electric field and 2D Poynting vector in the *y*-*z* plane in different layers in the *x*-direction when *θ* = 45°, *κ* = +0.4 and the beam is s-polarization. It is unambiguous that the momentum has a bias to the +*y* direction in most of the layers, resulting in *F*_y_ < 0.

(a)

(b)


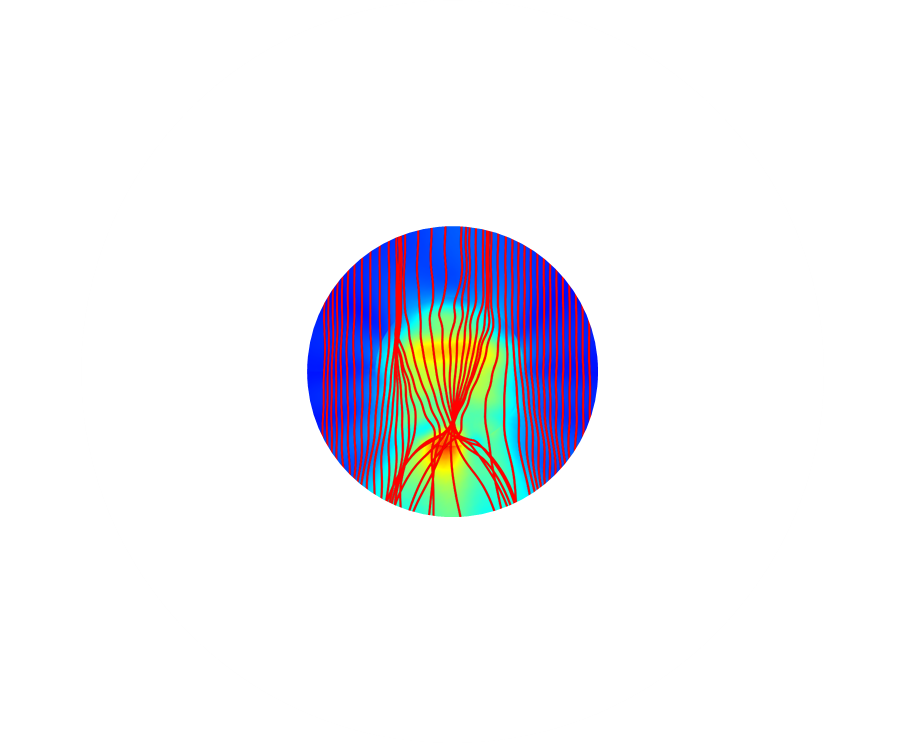


*y*

*z*

s, 10°

(c) δ = 0

(d) δ = 60 nm

(e) δ = 120 nm

(f) δ = 180 nm


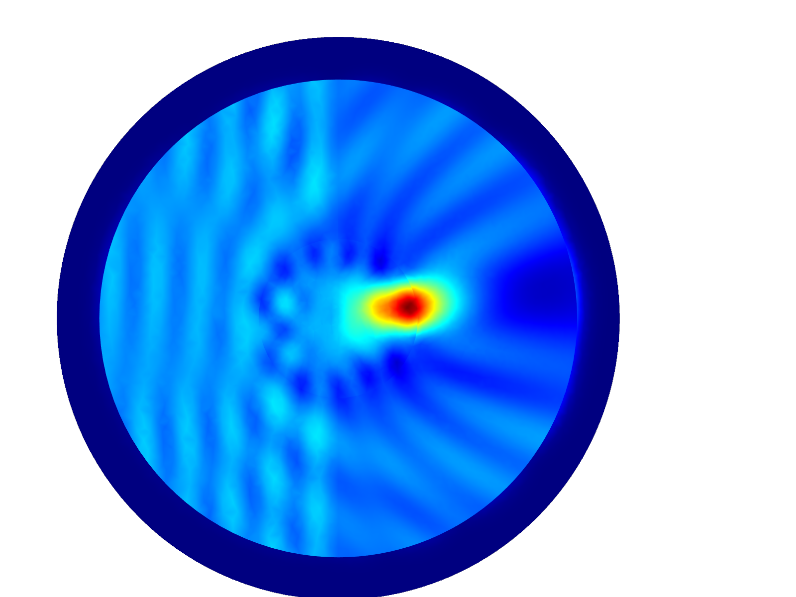


*x*

*z*

δ


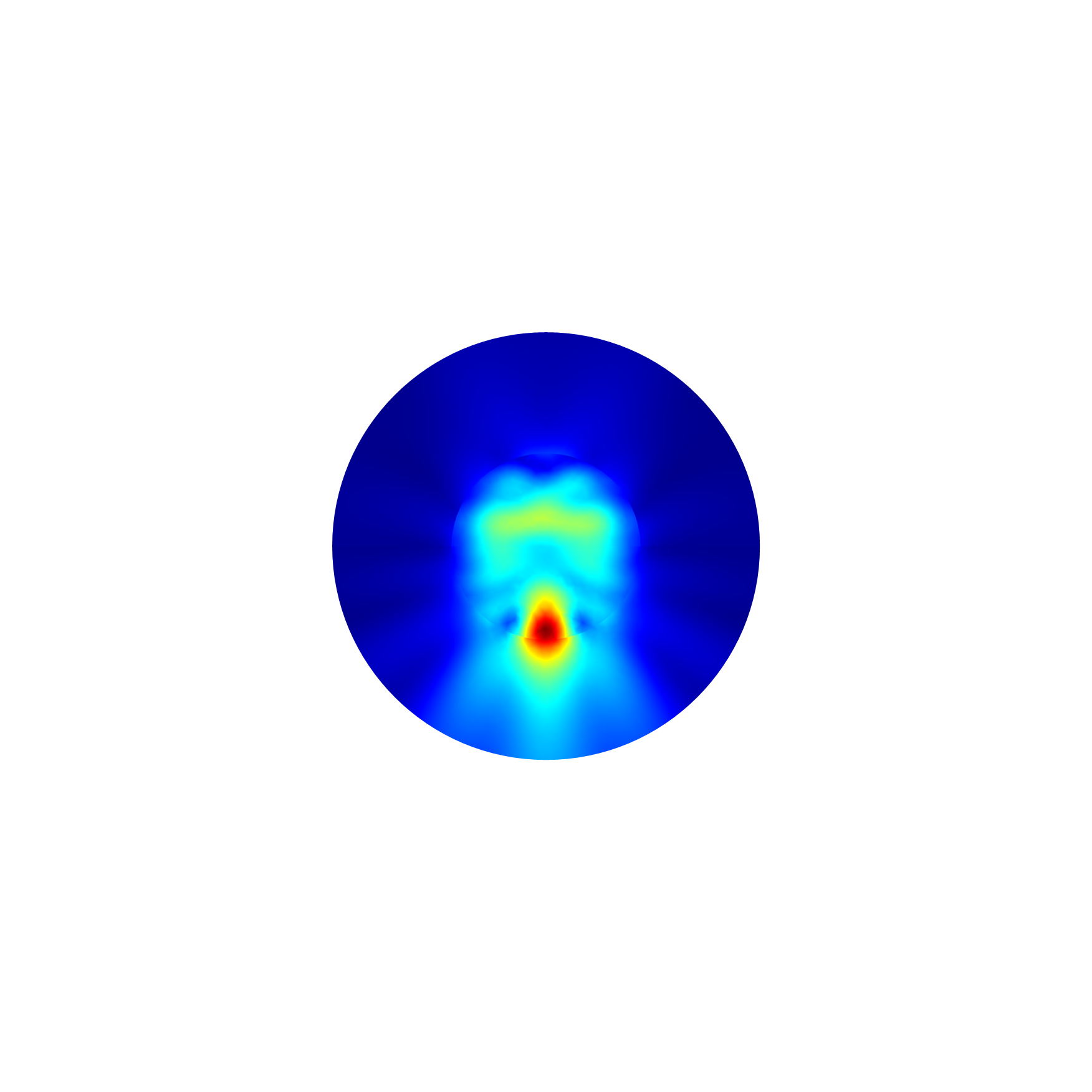

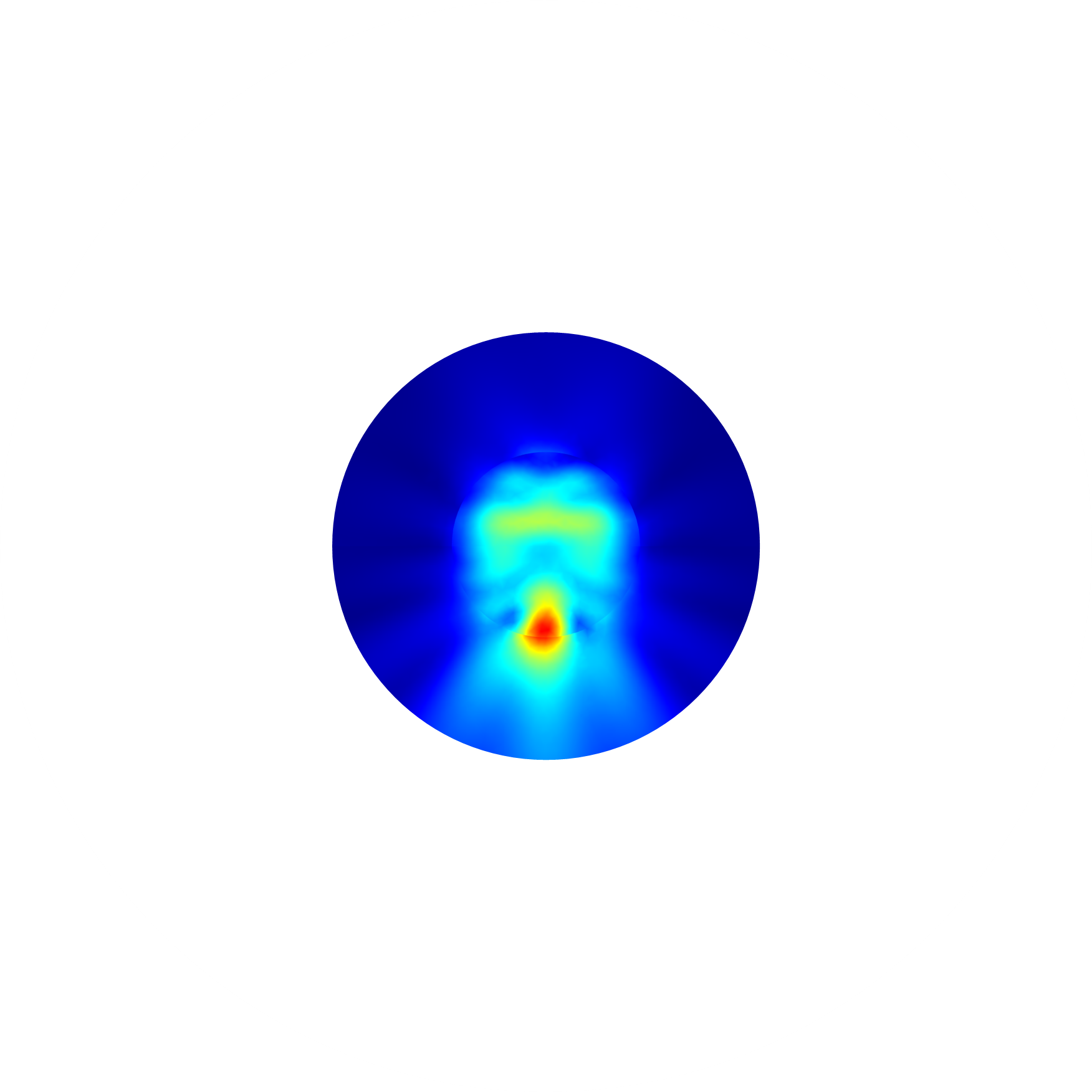

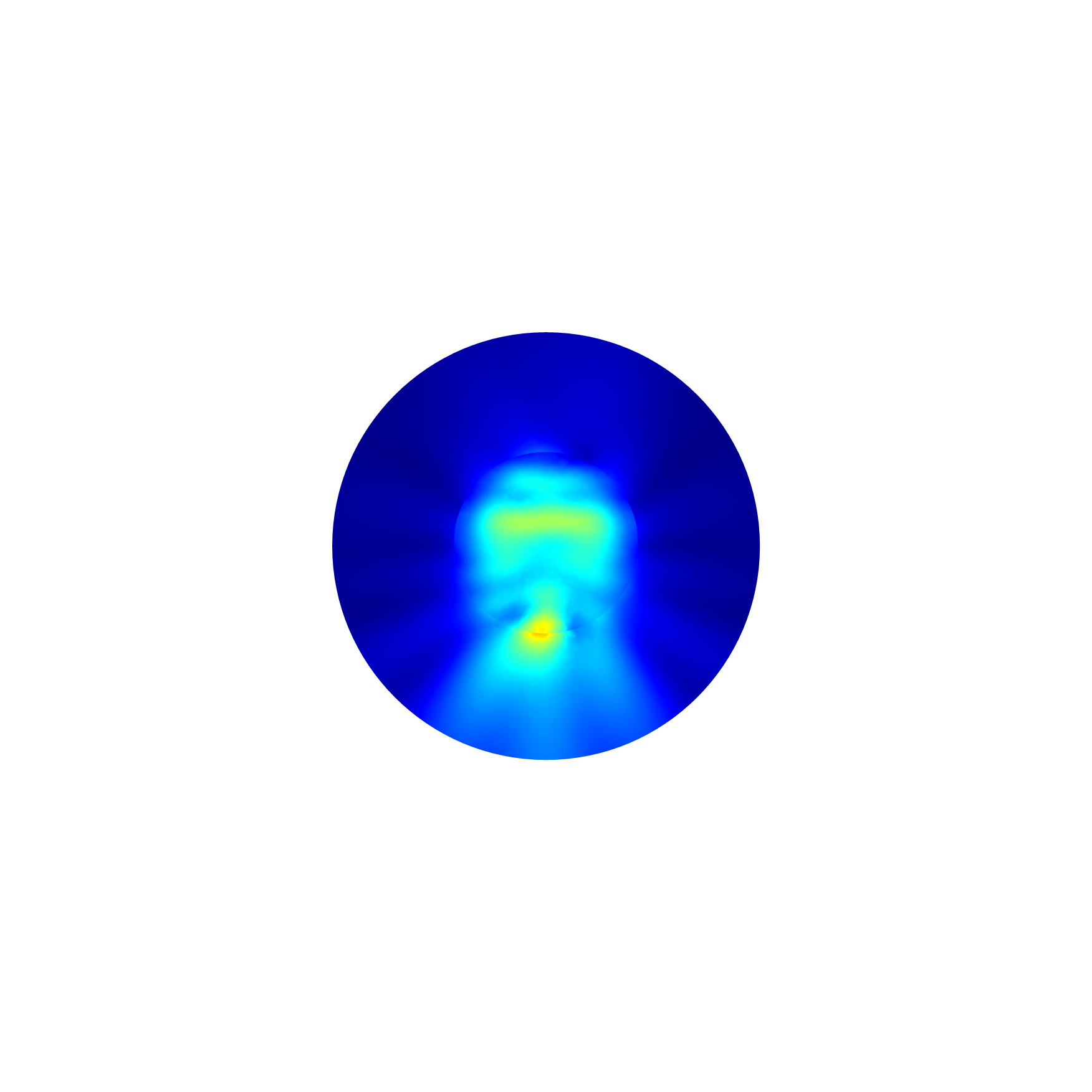

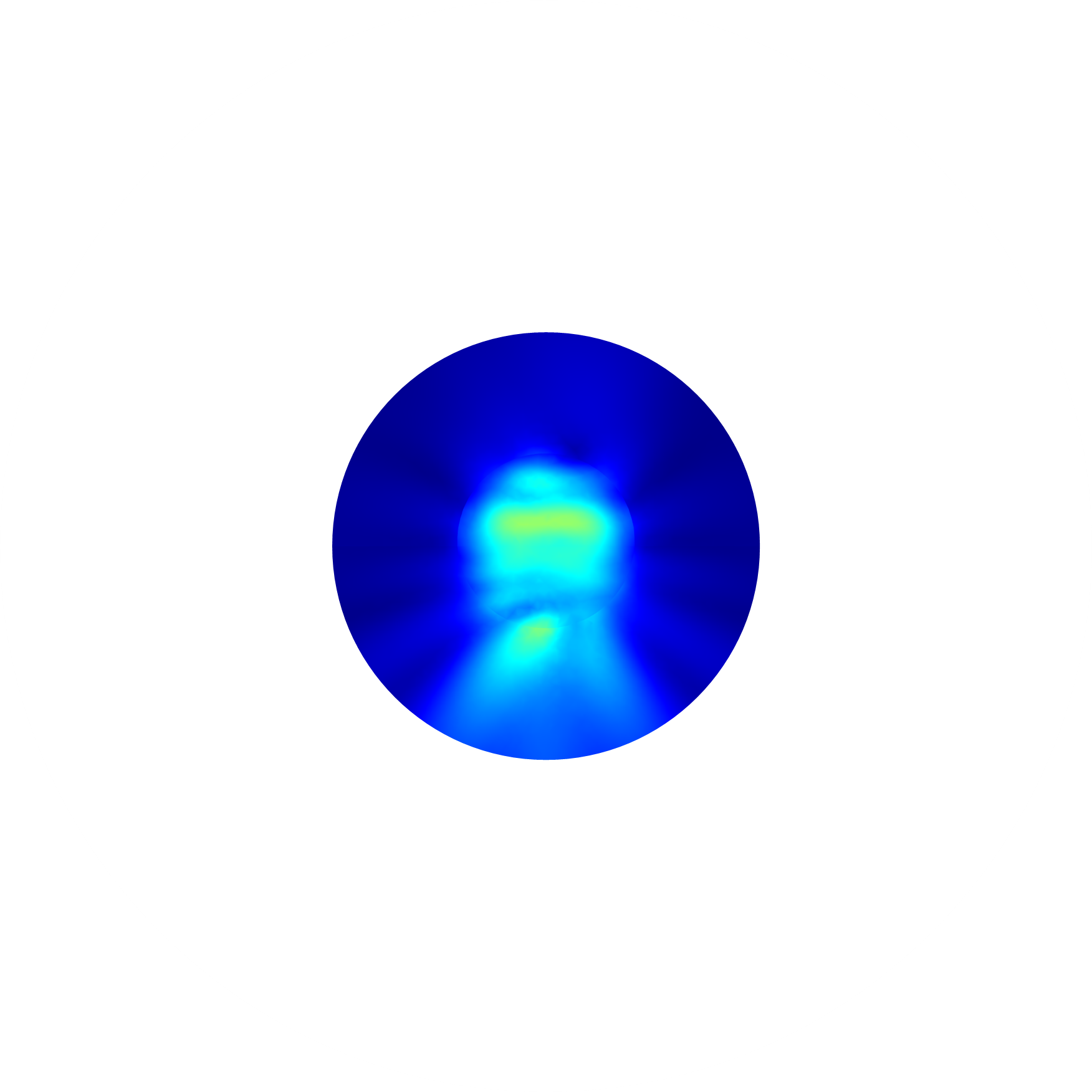


0


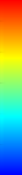


1

Fig. S6. Simulation of the optical field and Poynting vector when the incident angle is 10°. (a) *y*-*z* projection of electric field and 3D Poynting vector of a chiral particle (*κ* = +0.4) under the illumination of an s-polarized beam with incident angle of 10°. (b) Normalized electric field in the *x*-*z* plane. (c)-(f) Plot of scattering field in different inclined planes with *δ* from 0 to 180 nm. All sub-figures share the same colour bar.

(a)

(b)

(c)

*y*

*z*

*z*


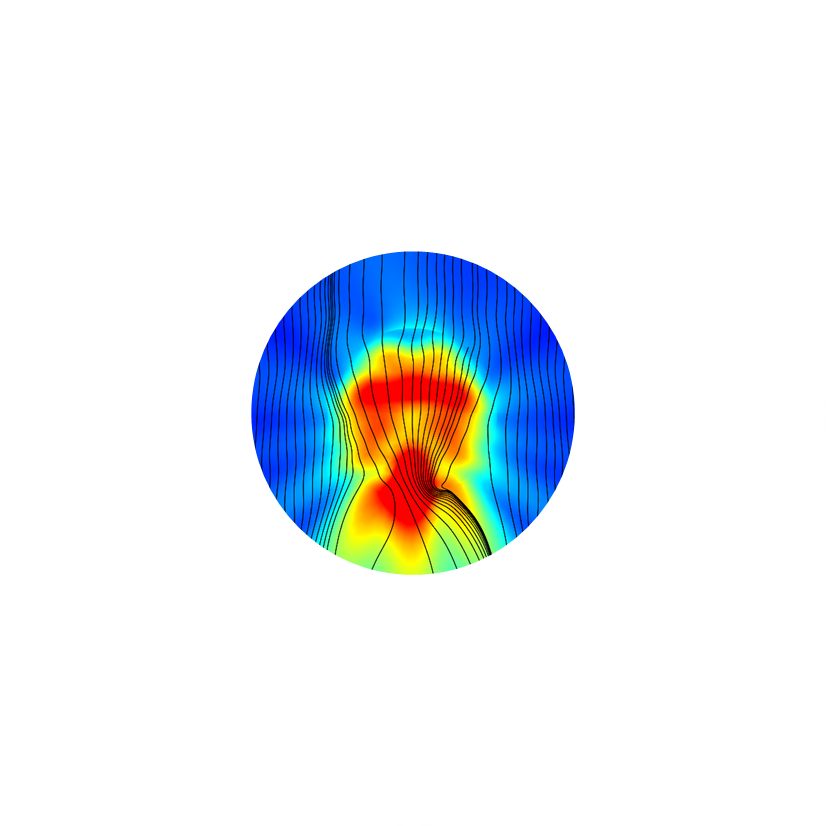


p, 10°


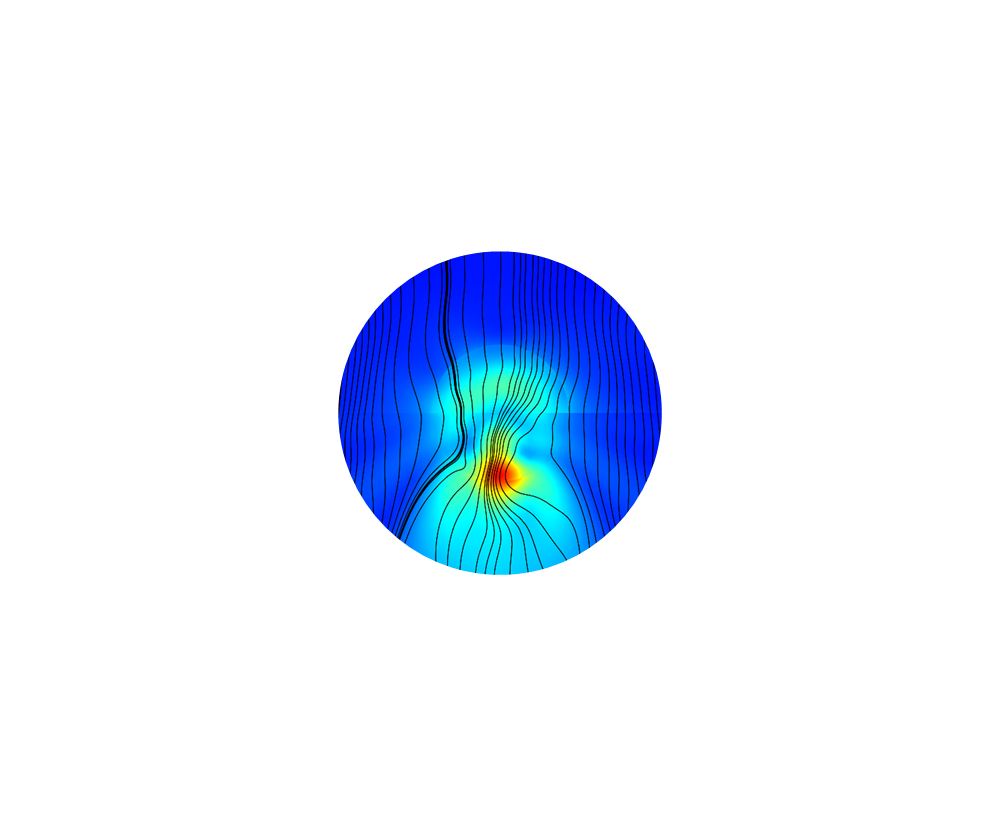


*y*

*z*

p, 45°


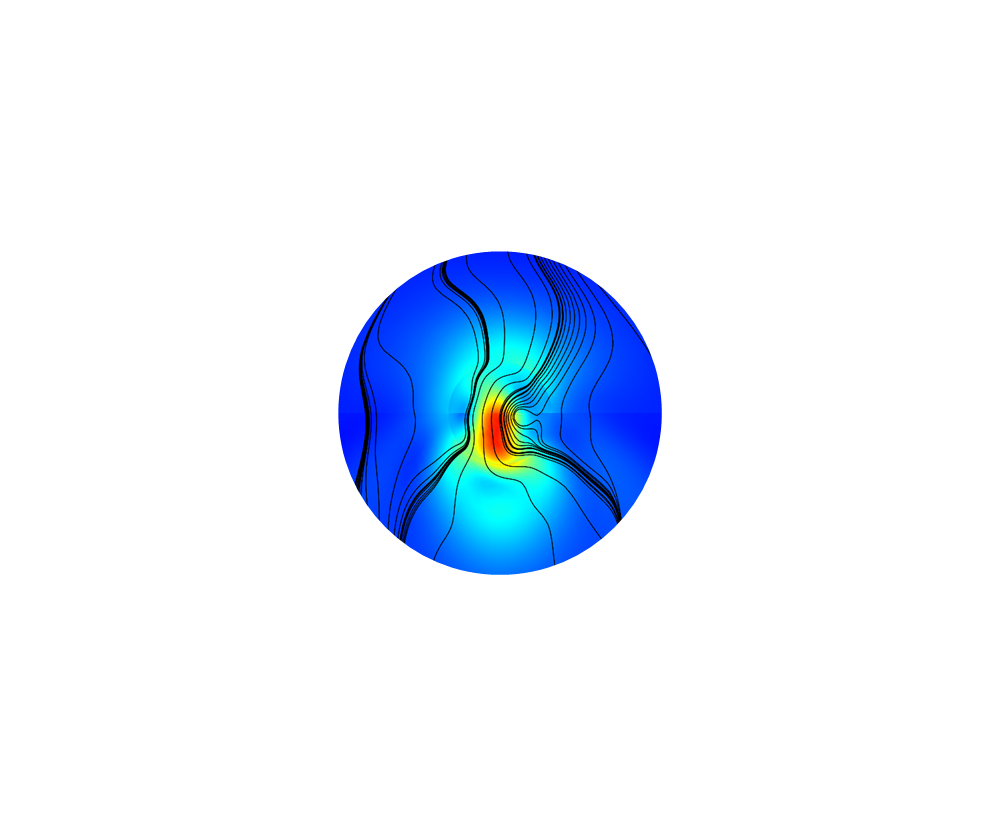


p, 80°


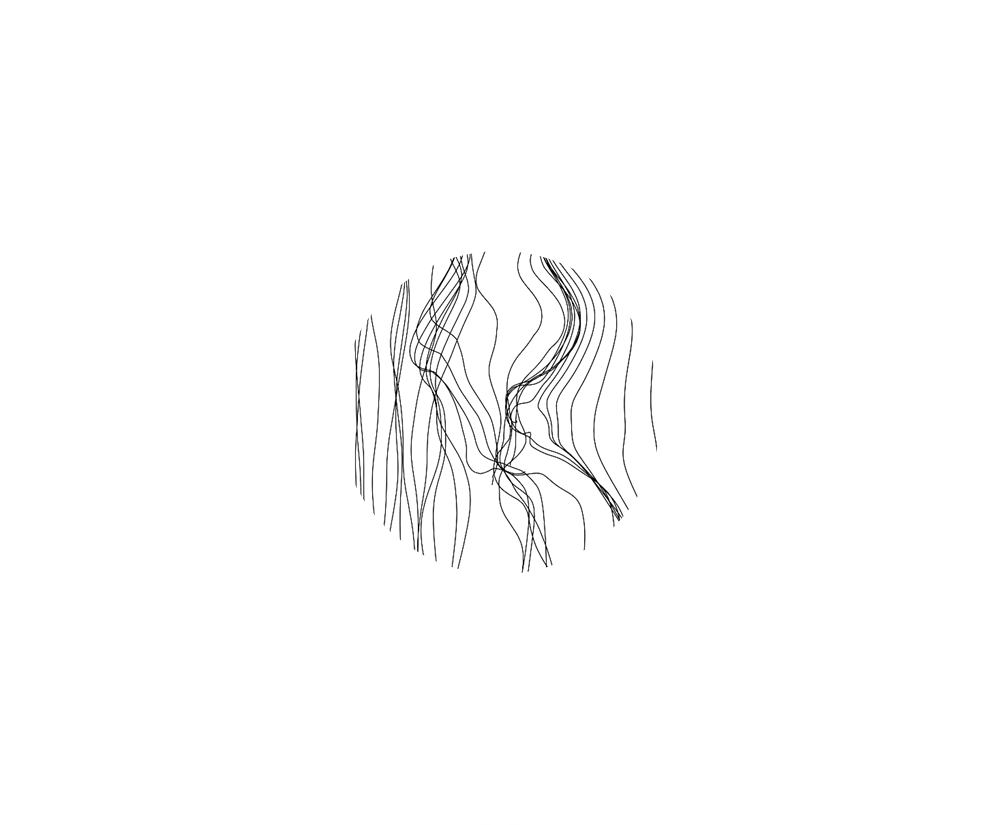


p, 80°

(d)

Fig. S7. Reversible optical lateral force on Mie chiral particles by the effect of incident angle for the p-polarization. Poynting vectors in the *y*-*z* planes for (a) 10°, (b) 45° and (c) 80° tend to point to (a) right, (b) left and (c) right, respectively. For a better view of the Poynting vector distributions, the *x* coordinates for the *y*-*z* planes in (a), (b) and (c) are 0, 300 nm and 400 nm, respectively. (d) The *y*-*z* projection (*x* = 0) of 3D Poynting vector of a chiral particle (*κ* = +0.4) under the illumination of p-polarized beam with incident angle of 80°. The overall distribution of the Poynting vector points to the right.


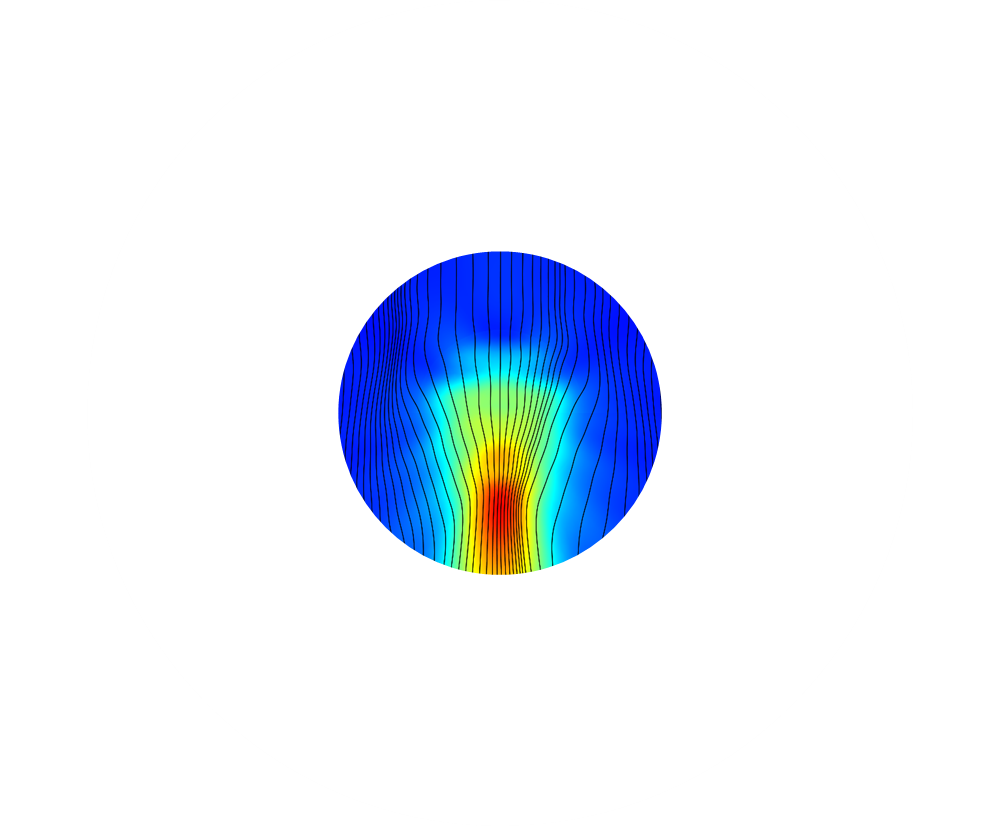

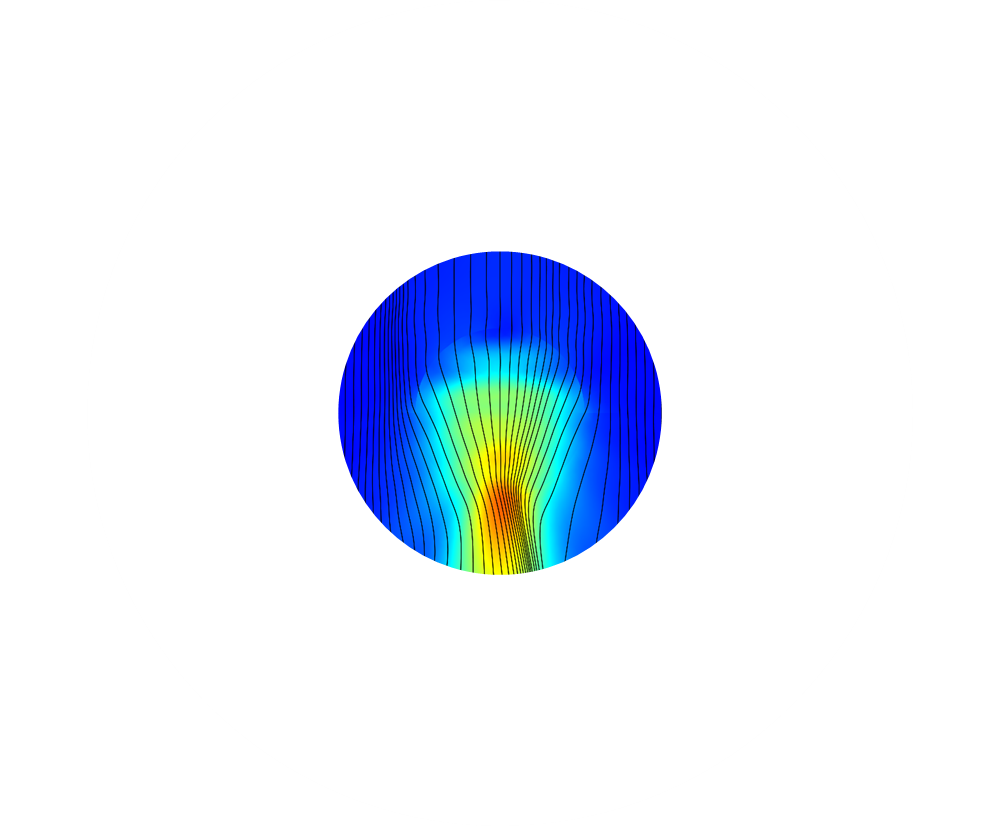


(a)

(b)

*y*

*z*

*z*

p, 10°, *κ* 0.1

*y*

*z*

s, 10°, *κ* 0.1

Fig. S8. Reversible optical lateral force on the 500 nm chiral particle when *κ* = 0.1. The Poynting vector distributions show the net left and right lateral forces for (a) the s- and (b) p-polarizations, respectively. The *x* coordinates of the *y*-*z* planes in (a) and (b) are 0 and 100 nm, respectively.


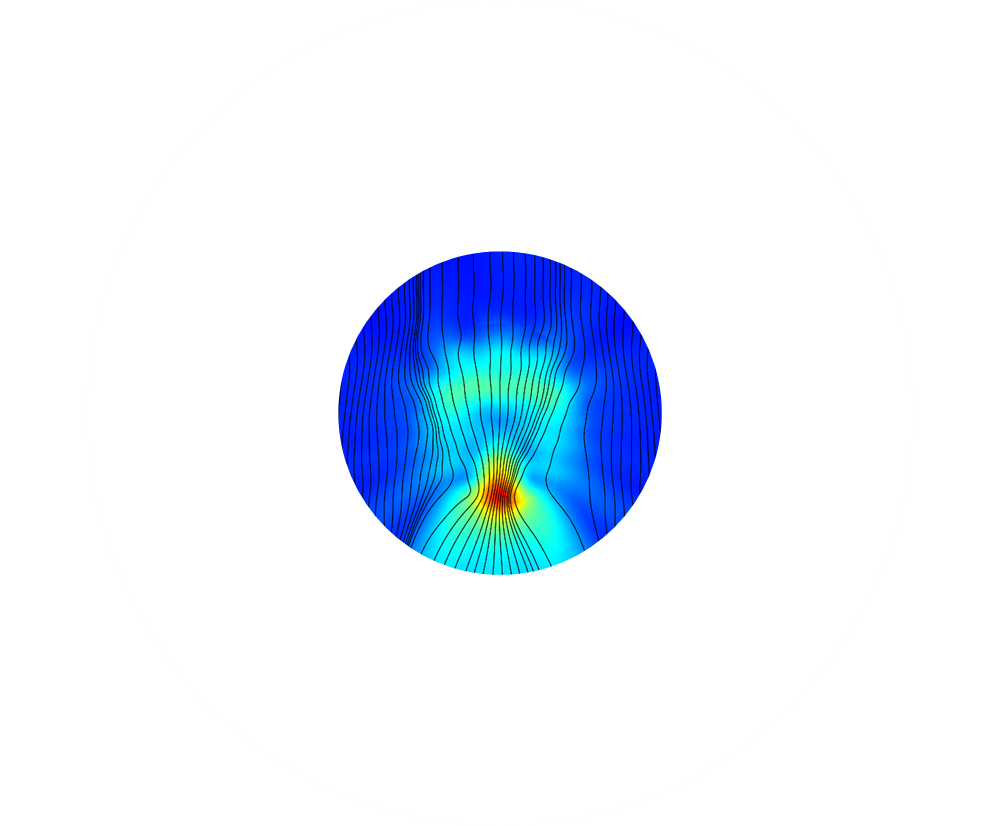

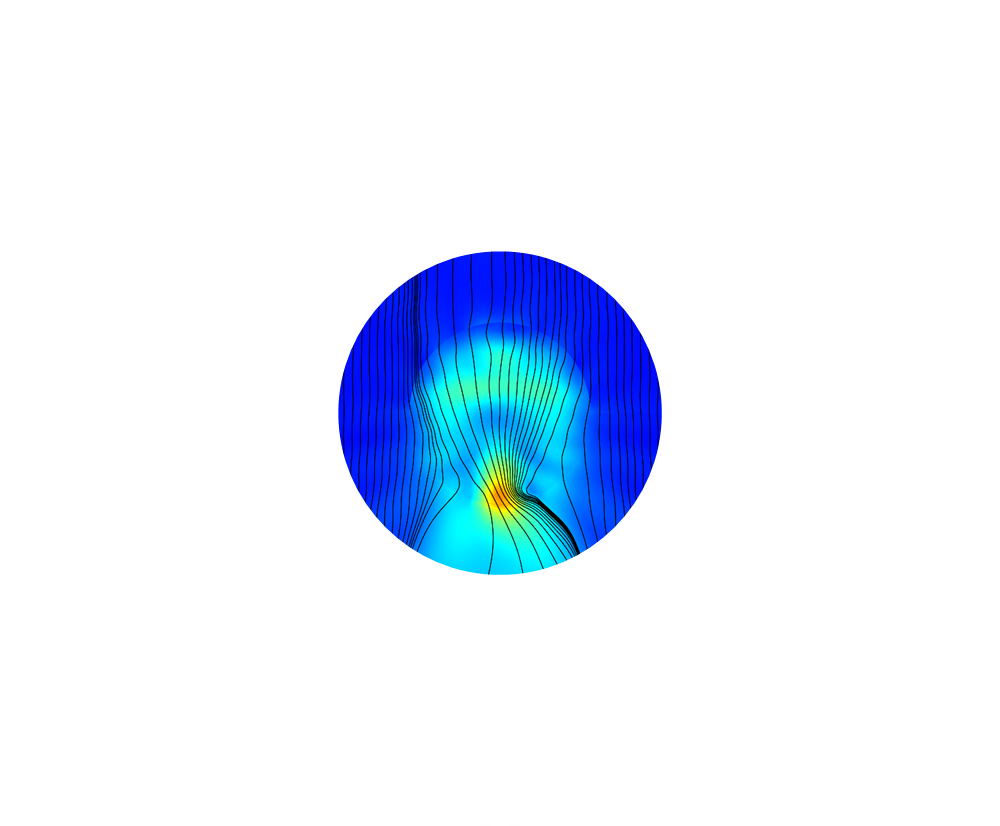


(a)

(b)

*y*

*z*

*z*

p, 10°, *κ* = 0.1

*y*

*z*

s, 10°, *κ* = 0.1

Fig. S9. Poynting vector distributions for 550 nm chiral particles. The 550 nm chiral particle (*κ* = 0.1) experiences (a) negative and (b) positive lateral forces for s- and p-polarizations, respectively. The incident angle is 10°. The *x* coordinates of the *y*-*z* planes in (a) and (b) are 0 and 100 nm, respectively.


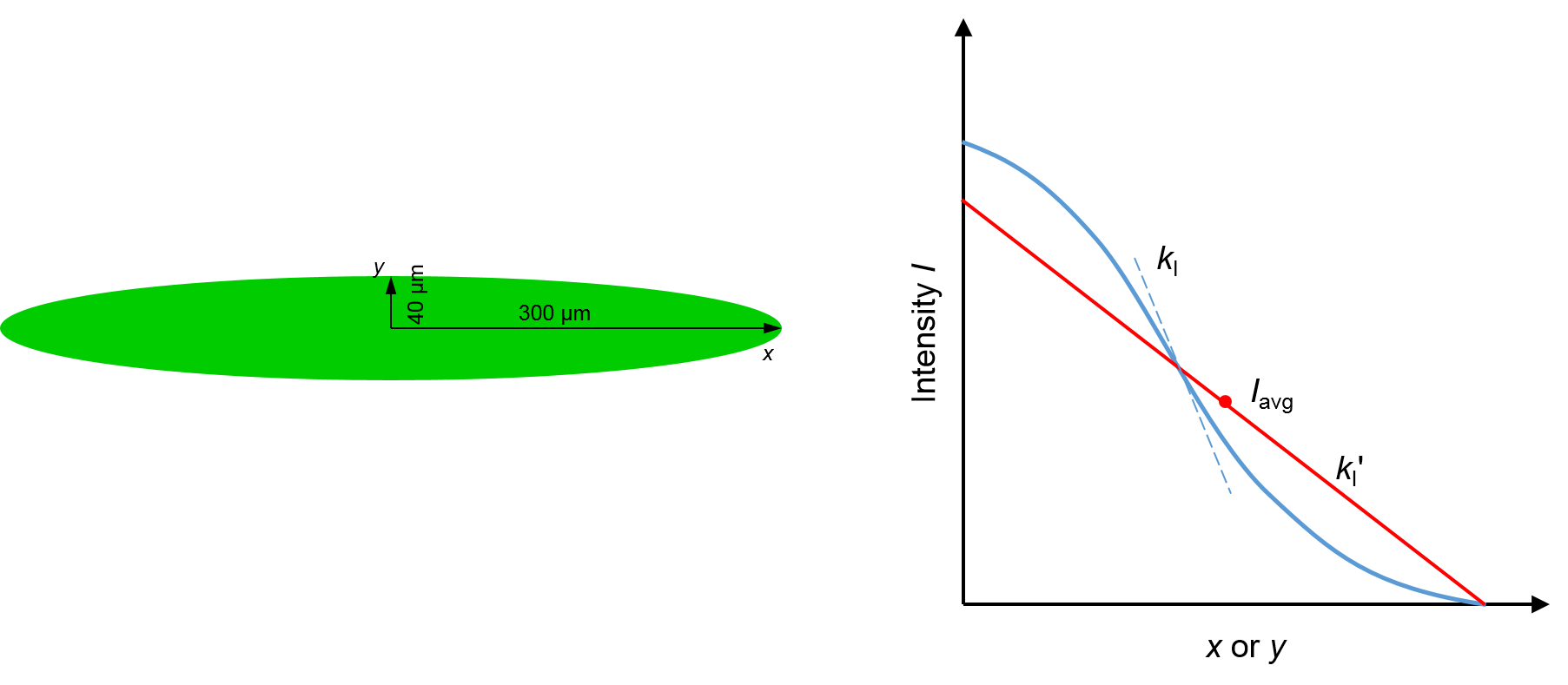


(a)

(b)

Fig. S10. Estimation of the intensity gradient in an ellipse hotspot. (a) Illustration of the laser spot. (b) Estimation of intensity gradient using a linear gradient scheme.

To estimate the intensity gradient, we first calculate the average intensity in the ellipse laser spot, which can be expressed as

*I*_avg_ = 1.4/(40×10^-6^×300×10^-6^π) = 3.7136×10^7^ (W/m^2^)

Then the intensity gradient can be estimated as

*k*_I_ = 3.7136×10^7^×4/300×10^-6^ = 4.95×10^11^ (W/m^3^)

For a normal optical trapping system, the intensity gradient is around

*k* = 10^9^ / 500×10^-9^ = 2×10^15^ (W/m^3^)

This intensity gradient in the experiment is much smaller than that in the conventional optical trapping system.

Because the electric field is set in COMSOL, we need to deduce the electric field for the intensity gradient configuration.

Let *I* = *k*_I_*x*,

Then $I=\frac{n^{2}c\varepsilon_{0}}{2}E^{2}=k_{I}x$,

$E=\sqrt{\left(\frac{2k_{I}x}{c\varepsilon_{0}n^{2}}\right)}\sqrt{x}=k_{E}\sqrt{x}=1.45\times10^{7}\sqrt{x}$,

Modifying the electric field of the obliquely incident beam in COMSOL, we get the simulation results in Fig. S11.


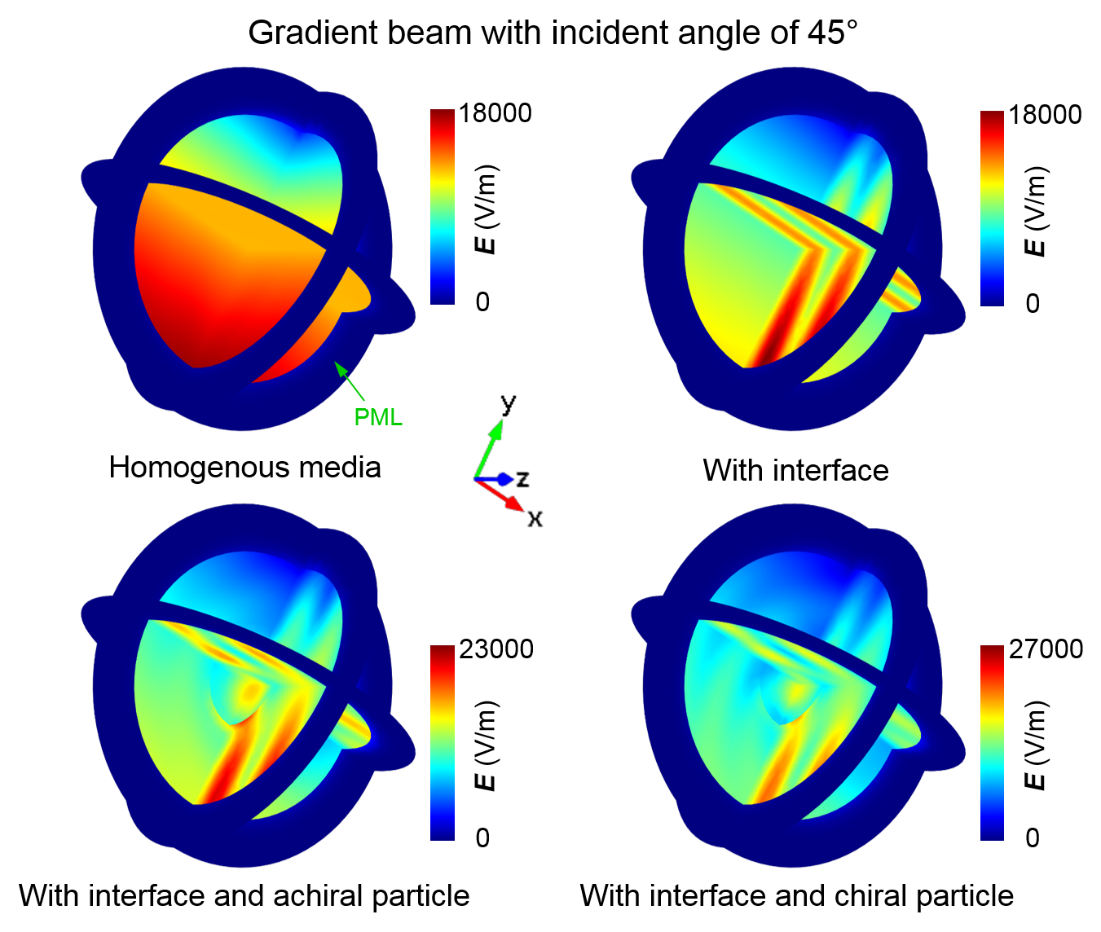


(a)

(b)

(c)

(d)

(e)

(f)

Fig. S11. Simulation of optical gradient and lateral forces in a gradient-intensity field. Illustration of obliquely incident light (a) in a homogenous medium and (b) at the interface of air and water. Electric field distribution when an (c) achiral or (d) chiral particle is placed at the interface. (e) Optical lateral force on the chiral particle, and optical gradient forces on the achiral particle in the *x*- and *y*-directions. The optical lateral force is at least one order of magnitude larger than the optical gradient force in the *x*-direction (used for the confine particles in the line trap). The optical lateral force is at least two orders of magnitude larger than the optical gradient force in the *y*-direction, which may be considered as the rival of the optical lateral force. (f) Optical lateral and gradient forces with the relation of *k_E_* in the line trap. The optical lateral force is at least two orders of magnitude larger than the optical gradient force in the condition of the same *k_E_*.


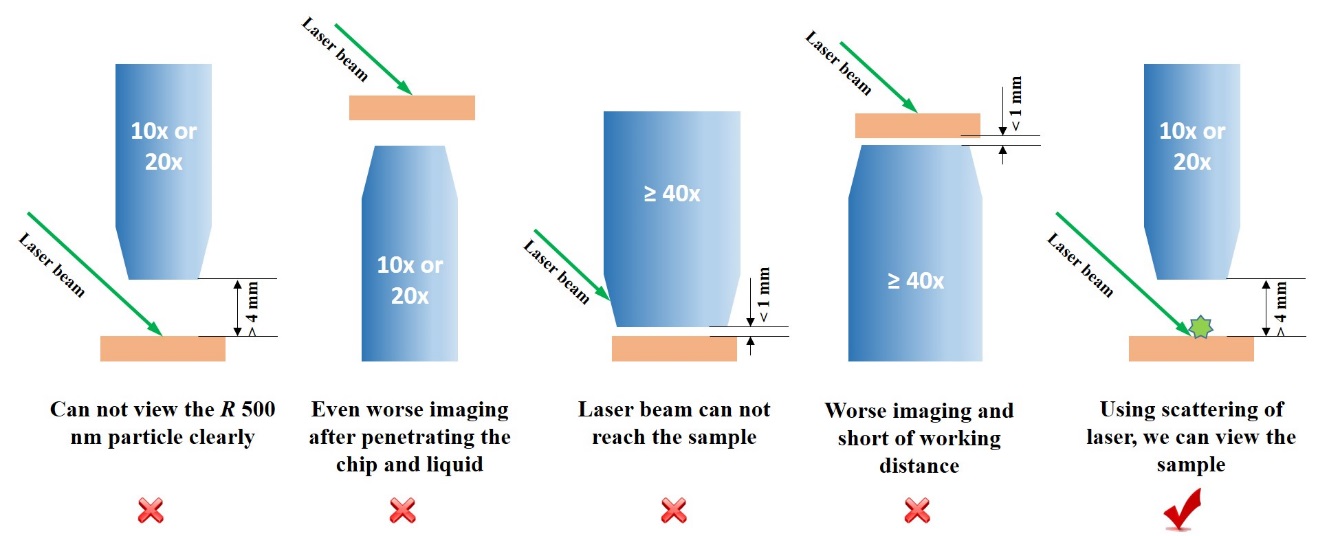


(a)

(b)

(c)

(d)

(e)


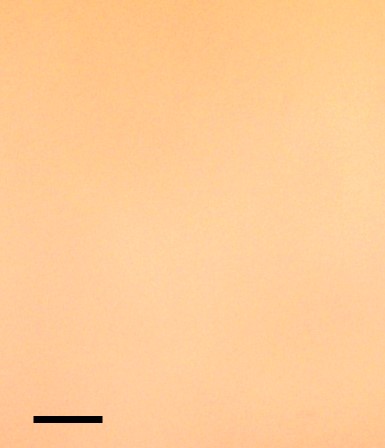

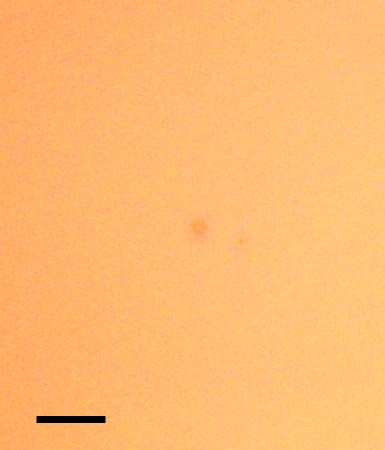


(f)

(g)

Fig. S12. Methods to view the chiral microparticles. Both (a) upright and (b) inverted microscope with a low power objective lens (10x or 20x) cannot view the 500 nm chiral particles clearly. (c) Laser beam cannot reach the sample when a high-power objective lens is on top because of the blocking of light. (d) The working distance of the high-power objective lens is not long enough to penetrate the chip and water to reach the sample in the inverted microscope. (e) A proper choice is to observer the scattering light from the chiral particle using a low power objective lens. Also, the low objective lens enables a much larger observation area. Bright field image of the chiral particle using upright (f) 10x and (g) 40x objective lens. The 10x objective lens cannot view any chiral particles. The 40x objective lens can only view few high-contrast chiral particles. While, most of the chiral particles including big particles cannot be observed. Based on the above discussion, the observation of chiral particles using the scattering light is an efficient and necessary approach.


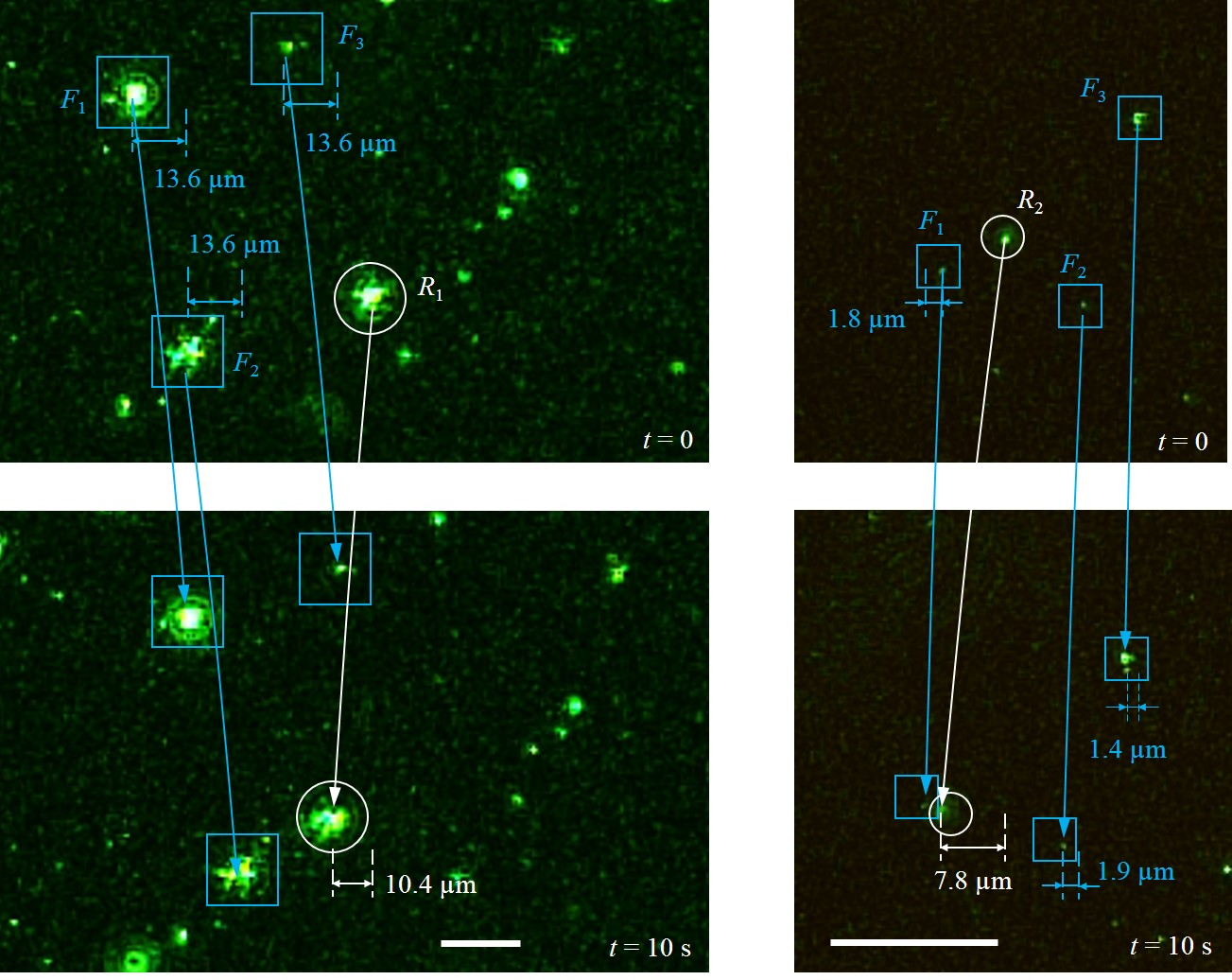


(a)

(b)

Fig. S13. Experimental results about the lateral movement of chiral particles and background flow. The background flow can have the (a) opposite and (b) same directions with the optical lateral forces. The velocities of the background flow in (a) and (b) are +1.36 µm/s and -0.14 µm/s, respectively. Chiral particles in (a) and (b) are both right handed (*κ* > 0). The scale bars in (a) and (b) equal 20 µm.


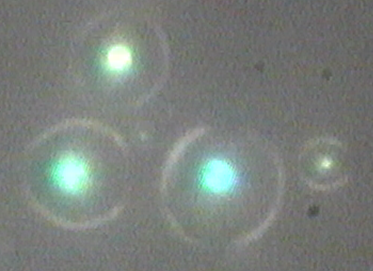

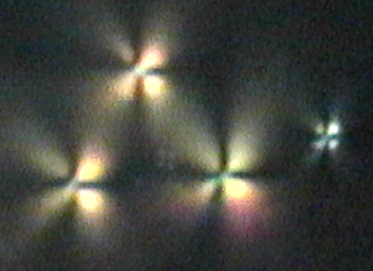


(a)

(b)

Fig. S14. Optical images of the chiral microparticles. The optical microscope images of the polymerized particles observed in (a) reflection and b) transmission modes. The slightly different color observed in reflection mode image supports that a small dispersion in the pitch exists in the particle suspension. Such dispersion can be justified by a non-uniform polymerization condition of the precursor droplets in the emulsions. The scale bars in (a) and (b) equal 2 µm.
